# Streptococcal autolysin promotes dysfunction of swine tracheal epithelium by interacting with vimentin

**DOI:** 10.1371/journal.ppat.1010765

**Published:** 2022-08-03

**Authors:** Yu Meng, Qing Wang, Zhe Ma, Weiyi Li, Kai Niu, Ting Zhu, Huixing Lin, Chengping Lu, Hongjie Fan

**Affiliations:** 1 MOE Joint International Research Laboratory of Animal Health and Food Safety, College of Veterinary Medicine, Nanjing Agricultural University, Nanjing, China; 2 Jiangsu Co-innovation Center for Prevention and Control of Important Animal Infectious Diseases and Zoonoses, Yangzhou University, Yangzhou, China; University of California, San Francisco, UNITED STATES

## Abstract

*Streptococcus suis* serotype 2 (SS2) is a major zoonotic pathogen resulting in manifestations as pneumonia and septic shock. The upper respiratory tract is typically thought to be the main colonization and entry site of SS2 in pigs, but the mechanism through which it penetrates the respiratory barrier is still unclear. In this study, a mutant with low invasive potential to swine tracheal epithelial cells (STECs) was screened from the TnYLB-1 transposon insertion mutant library of SS2, and the interrupted gene was identified as autolysin (*atl*). Compared to wild-type (WT) SS2, Δ*atl* mutant exhibited lower ability to penetrate the tracheal epithelial barrier in a mouse model. Purified Atl also enhanced SS2 translocation across STEC monolayers in Transwell inserts. Furthermore, Atl redistributed the tight junctions (TJs) in STECs through myosin light chain kinase (MLCK) signaling, which led to increased barrier permeability. Using mass spectrometry, co-immunoprecipitation (co-IP), pull-down, bacterial two-hybrid and saturation binding experiments, we showed that Atl binds directly to vimentin. CRISPR/Cas9-targeted deletion of vimentin in STECs (VIM KO STECs) abrogated the capacity of SS2 to translocate across the monolayers, SS2-induced phosphorylation of myosin II regulatory light chain (MLC) and MLCK transcription, indicating that vimentin is indispensable for MLCK activation. Consistently, vimentin null mice were protected from SS2 infection and exhibited reduced tracheal and lung injury. Thus, MLCK-mediated epithelial barrier opening caused by the Atl-vimentin interaction is found to be likely the key mechanism by which SS2 penetrates the tracheal epithelium.

## Author summary

*Streptococcus suis* serotype 2 (SS2), an emerging zoonotic agent, can breach the respiratory barrier and cause invasive disease in pigs. Here, we identified the novel role of autolysin Atl in penetration of the respiratory barrier by SS2 and its systemic dissemination and identified its binding partner, vimentin, a type III intermediate filament protein. Atl contributed to the MLCK-triggered redistribution of tight junctions to open the tracheal epithelial barrier. Knockout of vimentin abolished the ability of SS2 to penetrate the monolayer barrier and the activation of MLCK. Furthermore, vimentin null mice were protected from infection by intranasally administered SS2. This study is the first to demonstrate that the interaction between the GBS Bsp-like domain of Atl and vimentin promotes MLCK-mediated dysfunction of the epithelial barrier, which may provide theoretical information for prophylactic and/or therapeutic treatments against diseases caused by similar respiratory pathogens.

## Introduction

*Streptococcus suis* is a Gram-positive opportunistic bacterium that asymptomatically colonizes in the upper respiratory tract and is divided into 29 different serotypes based on capsular polysaccharide antigens [1, 2]. *S*. *suis* serotype 2 (SS2) is the serotype with the highest clinical isolation rate and is regarded as one of the most virulent in pigs and humans [3, 4]. SS2 usually infects pigs through the respiratory tract, causing sepsis and pneumonia and thereby significantly endangers the development of the pig industry worldwide [5, 6]. Through contaminated pork or close contact with infected pigs, humans can also be infected with SS2, which mainly manifests as streptococcal toxic shock-like syndrome (STSLS) and meningitis [4, 7]. Indeed, SS2 infection is the primary cause of human meningitis in Southeast Asia, especially Vietnam and Thailand [8].

Respiratory tract pathogens exploit various strategies to penetrate the epithelial barrier, enter the bloodstream and cause systemic infection [9, 10]. The respiratory epithelium of the upper respiratory tract forms a physical barrier to defend against various pathogens and harmful substances inhaled from the external environment [11, 12]. Tight junctions (TJs) and adhesion junctions (AJs) located at points of cell-cell contact seal the paracellular space and are predominant determinants of paracellular permeability [13, 14]. The tight junction adaptor protein, Zonula occludens (ZOs), connect the cytoplasmic regions of the transmembrane proteins, occludin and claudin, with the actin cytoskeleton to maintain the structure and function of TJs [15]. Myosin II belongs to the myosin superfamily which are actin-based molecular motors involved in diverse motility-related cellular activities [16]. Myosin II regulatory light chain (MLC) and actin cytoskeleton form the perijunctional actomyosin ring. Activated myosin light chain kinase (MLCK) can phosphorylate MLC, resulting in remodeling of TJs and increased paracellular permeability [17].

Bacteria possess a variety of peptidoglycan hydrolases (also known as autolysins), which are indispensable for cell wall peptidoglycan turnover during growth, separation of daughter cells, and autolysis [18, 19]. Peptidoglycan hydrolases are classified into 1) glycosidases, which cleave glycan backbones, 2) amidases, which cleave side-chain peptides, and 3) peptidases (endopeptidases and carboxypeptidases), which cleave within the peptide side-chains [20]. In addition to these peptidoglycan hydrolase activities, autolysins have also been reported to be involved in bacterial pathogenicity [21–23].

In this study, an *atl* mutant associated with SS2 penetration of the tracheal epithelial barrier was screened from the TnYLB-1 transposon insertion library of SS2. We demonstrated that Atl contributes to SS2 penetration of the tracheal epithelium and systemic dissemination in mice. Furthermore, we showed that Atl induces the redistribution of TJs in swine tracheal epithelial cells (STECs) through the MLCK pathway, resulting in increased barrier permeability. Additionally, we found that the interaction between Atl and the cytoskeletal component vimentin contributes to SS2 penetration of the tracheal epithelial barrier.

## Results

### Atl contributes to SS2 penetration of the tracheal barrier and systemic dissemination

Qualitative and quantitative screening of the TnYLB-1 transposon insertion mutant library of ZY05719 was performed to identify SS2 genes that are associated with penetration of the tracheal epithelial barrier [24]. Among the 13 mutants with reduced invasion ability, the invasion ability of the *atl* mutant was significantly reduced (S1 Fig and S1 Table). To verify the functions of *atl*, the *atl*-deleted strain Δ*atl* was constructed by electroporation of the Δ*atl*-pSET4S plasmid into ZY05719, and CΔ*atl*-pSET4S was electroporated into Δ*atl* to construct the in situ complementary strain CΔ*atl* (S2 Fig).

**Fig 1:**
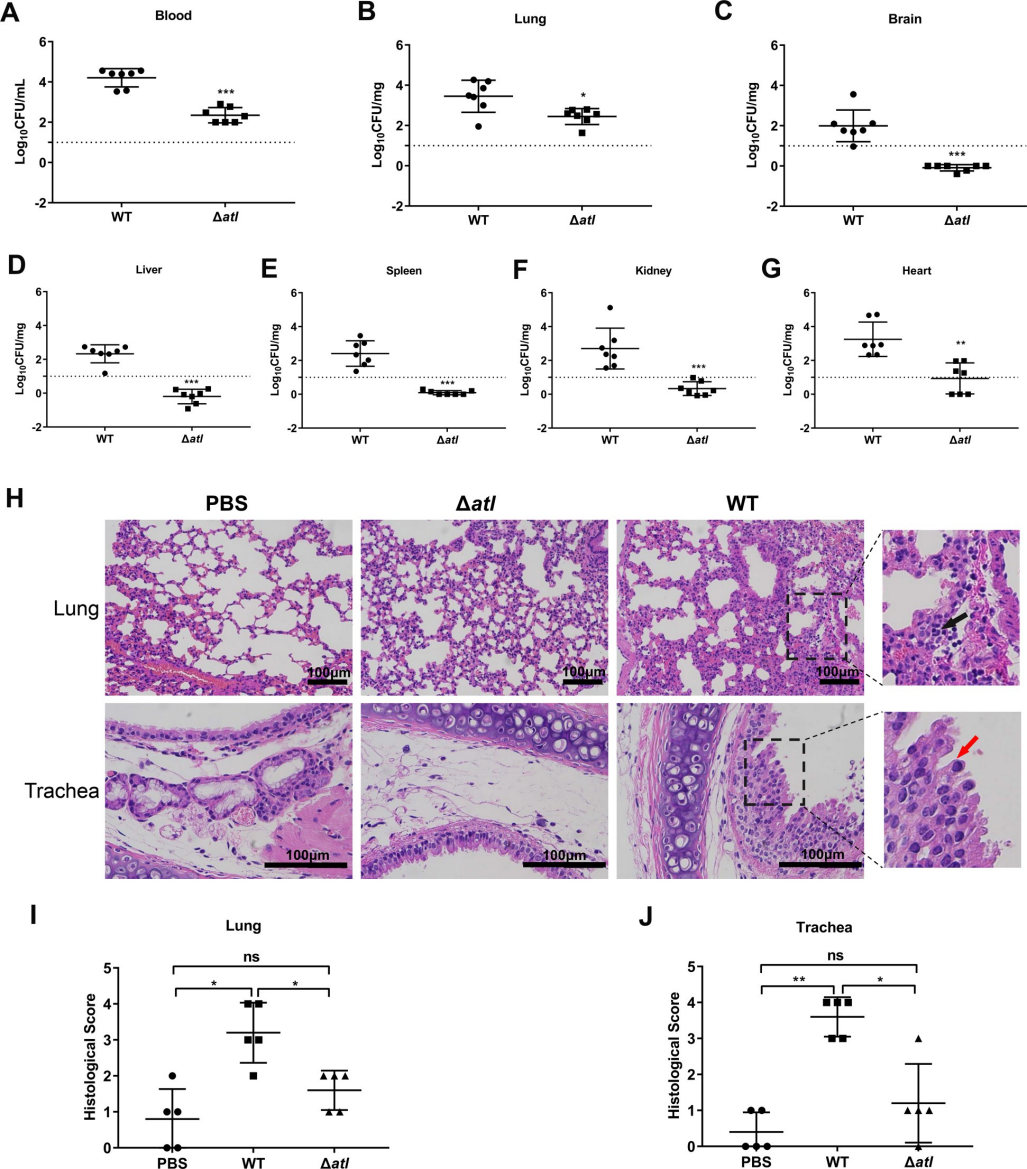
Atl contributes to penetration and systemic dissemination of SS2 in mice. (A-G) Bacterial loads in the blood (A), lung (B), brain (C), liver (D), spleen (E), kidney (F) and heart (G) of BALB/c mice intranasally challenged with 1 × 10^9^ CFUs WT SS2 or Δ*atl* mutant at 24 hpi (n = 7 mice/group). (H) H&E staining of tracheal and lung tissue sections from PBS-, WT SS2- or Δ*atl* mutant strain-infected mice by intranasal inoculation at 24 hpi. In the enlarged area of the images (black dashed frame), the “black arrow” indicates infiltration of inflammatory cells, and the “red arrow” indicates areas of translocated tracheal epithelium. Scale bar, 100 μm. (I-J) Pathological analysis of lungs (I) and trachea (J) by blinded assessment of H&E stained sections. The data shown are representative or are presented as the mean ± SD of the values obtained in three independent experiments. Statistical analysis was performed using Mann-Whitney test (A-G, I-J). ns, not significant; *, *P* < 0.05; **, *P* < 0.01; ***, *P* < 0.001.

Comparison of the ability of WT and Δ*atl* to penetrate the mouse tracheal epithelial barrier at 24 hpi showed that the colony-forming units (CFUs) in the blood of mice infected with WT SS2 were more than 100 times higher than those in the blood of mice challenged with Δ*atl* (Fig 1A). In addition, the CFUs in the lungs, brains, livers, spleens, kidneys, and hearts of mice infected with WT SS2 were also significantly higher than those in the lungs, brains, livers, spleens, kidneys, and hearts of mice infected with Δ*atl* (Fig 1B–1G).

Histopathological analysis of tracheal and lung tissues from mice challenged with WT SS2 revealed obvious peribronchial infiltration of inflammatory cells, thickened alveolar walls, and collapsed alveoli; the tracheal epithelium of the infected mice was translocated, and the epithelial cilia were damaged substantially (Fig 1H). The tracheal and lung tissues of the Δ*atl*-infected mice exhibited normal physiological structure similar to that in the control group (Fig 1H–1J). These results demonstrate that Atl promotes penetration of SS2 into the mouse respiratory tract and systemic dissemination.

### Atl induces TJ redistribution to increase tracheal epithelial barrier permeability

**Fig 2:**
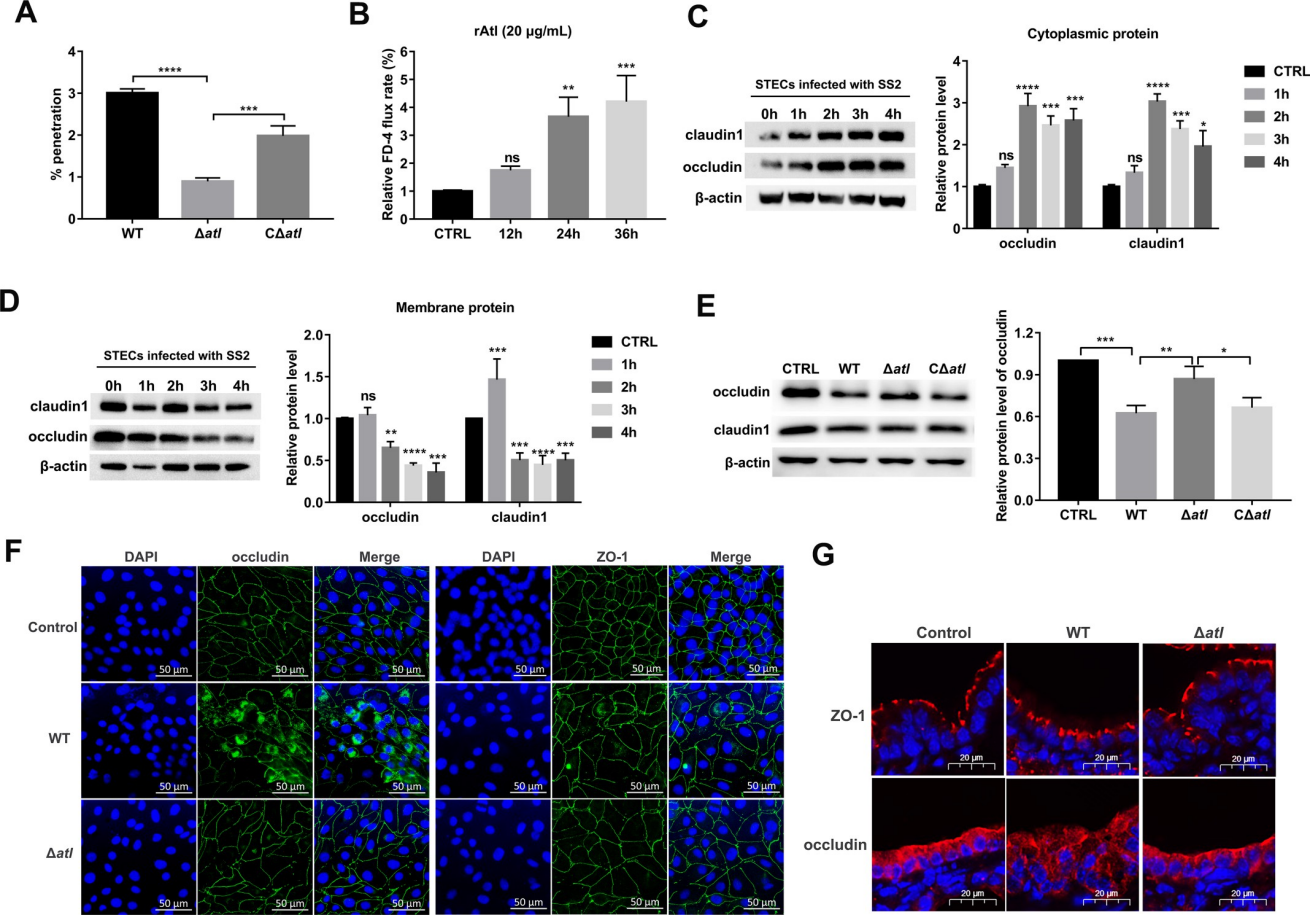
Atl contributes to the redistribution of TJs. (A) STEC monolayers on Transwell inserts were infected with WT, Δ*atl*, or CΔ*atl* at an MOI of 50:1. Penetration rates of WT, Δ*atl*, and CΔ*atl* from the apical side of Transwell inserts to the basolateral side were calculated at 3 hpi. (B) rAtl increases the permeability of the tracheal epithelial barrier model. STEC monolayers on the Transwell were incubated with endotoxin free rAtl (20 μg/mL) for 12 h, 24 h, and 36 h, and the FD-4 (1 mg/mL) flux into the basolateral chamber was then measured. The FD-4 flux in the control group was set as 1%, and the FD-4 flux in the rAtl-treated groups was compared with that in the control group. (C and D) STECs were infected with WT SS2 (MOI 50:1). The subcellular distribution of occludin and claudin1 in the cytoplasm (C) and cell membrane (D) from STECs was determined by Western blot at the indicated time points postinfection. Band intensity relative to the uninfected group was analyzed. (E) Effect of Atl on the distribution of claudin1 and occludin in the cell membrane of STECs. STECs were infected with WT, Δ*atl*, or CΔ*atl* at an MOI of 50:1. The expression of claudin1 and occludin in the cell membrane of STECs was analyzed by Western blot, and band intensity relative to the control was calculated. (F) Immunofluorescence images of occludin and ZO-1 (green) in STECs infected with WT SS2 or the Δ*atl* mutant. The cell nuclei were stained with DAPI (blue). Scale bar, 50 μm. (G) Immunofluorescence staining of ZO-1 and occludin (red) in lung bronchioles of BALB/c mice at 24 hpi after infection with WT SS2 or with the Δ*atl* mutant strain. The nuclei of bronchiolar epithelial cells were stained with DAPI (blue). Scale bar, 20 μm. The data shown are representative or are presented as the mean ± SD of the values obtained in three independent experiments. Statistical analysis was performed using one-way ANOVA with Dunnett’s multiple comparison test (A-B) or Tukey’s multiple comparison test (E) and two-way ANOVA with Dunnett’s multiple comparison test (C-D). ns, not significant; *, *P* < 0.05; **, *P* < 0.01; ***, *P* < 0.001; ****, *P* < 0.0001.

An *in vitro* tracheal epithelial barrier model using Transwell inserts was constructed to compare the abilities of WT SS2, Δ*atl*, and CΔ*atl* to penetrate the barrier (S3 Fig). Consistent with the results of the mouse experiment, the ability of Δ*atl* penetrated from the apical side to the basolateral media of tracheal epithelial barrier were significantly less than those of WT and CΔ*atl* (Fig 2A). The transepithelial electrical resistance (TEER) of the STEC monolayer challenged with WT or CΔ*atl* was reduced to 60%, significantly lower than that of the Δ*atl*-infected cells (S4 Fig). There was no significant difference in the growth of WT, Δ*atl* mutant, and CΔ*atl* strains under the same culture conditions (S5 Fig). Thus, the deficient barrier penetration ability of the Δ*atl* mutant was not due to a compromised bacterial growth rate. Moreover, the FD-4 permeability of recombinant Atl protein (rAtl)-treated STEC monolayers on Transwell inserts showed a time-dependent increase compared with that of PBS-treated monolayers (Fig 2B). These findings suggest that Atl affects the permeability of the tracheal epithelial barrier model.

Bacterial penetration of the barrier is associated with increased barrier permeability, and TJs play a crucial role in regulating that permeability. The effects of SS2 on the transcription and expression of the TJ proteins ZO-1, occludin, and claudin1 in STECs were determined by quantitative RT-PCR (qRT-PCR) and Western blot. The results showed that SS2 had no effects on the transcription of the genes that encode these proteins (S6A Fig) or on the expression levels of these TJ proteins over the time course of infection (S6B Fig). In addition, cell viability assays showed that neither WT nor Δ*atl* affected cell viability (S7 Fig).

The time-dependent distribution of TJs in detergent-soluble (cytoplasmic) and detergent-insoluble (cell membrane) fractions of STECs infected with SS2 was subsequently analyzed. At 3 hpi, the levels of claudin1 and occludin in the cytoplasm of STECs increased significantly, while the levels of these proteins in the cell membrane decreased (Fig 2C and 2D). However, the occludin content of the cell membranes of STECs infected with Δ*atl* was significantly higher than that of the cell membranes of cells infected with WT SS2 (Figs 2E and S8).

Immunofluorescence was performed to further investigate the distribution of TJ proteins in the cells. In the control cells, ZO-1 and occludin were evenly distributed at the cell boundary; in the WT infection group, ZO-1 and occludin delocalized from the apical junctions, and an increase in cytosolic occludin was observed (Fig 2F). In the Δ*atl* infection group, similar to that observed in the control cells, there was a slight disturbance of ZO-1 and occludin (Fig 2F). Immunofluorescence staining of mouse bronchioles confirmed the redistribution of ZO-1 and occludin in mice challenged with WT SS2, but not Δ*atl* (Fig 2G). Collectively, these observations suggest that Atl contributes to the redistribution of ZO-1 and occludin without affecting TJ protein expression during SS2 infection.

### Atl contributes to the activation of the MLC to redistribute TJs

**Fig 3:**
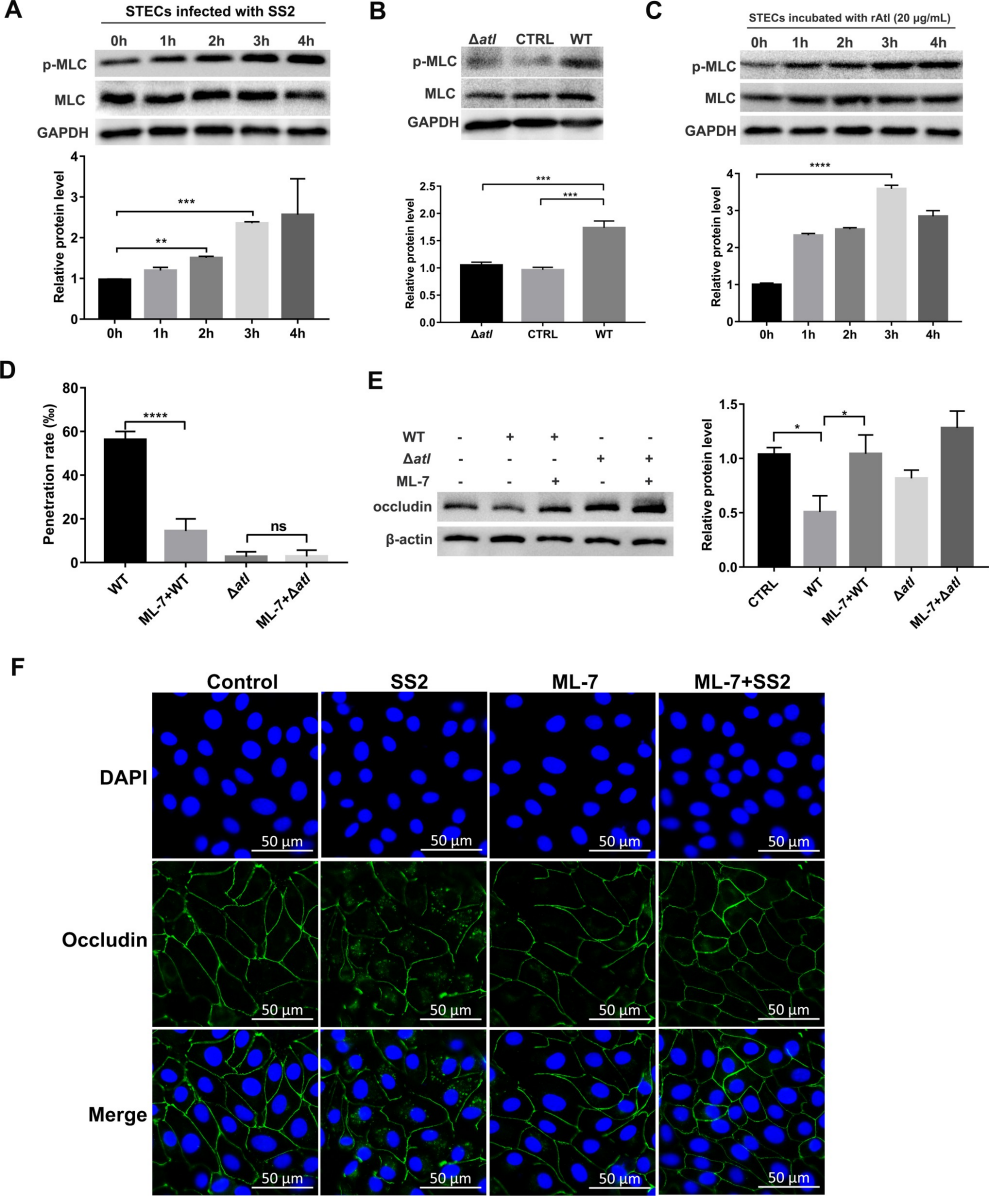
Atl contributes to the phosphorylation of MLC to redistribute TJs. (A) STECs were infected with WT SS2 (MOI 50:1) for the indicated times, and cell lysates were subjected to Western blot to assess the expression of phospho-MLC and MLC. Band intensity relative to the uninfected group was analyzed. (B) STECs were infected with WT SS2 or with the Δ*atl* mutant strain, and the expression of phospho-MLC and MLC was analyzed by Western blot at 3 hpi. Band intensity relative to the control was analyzed. (C) STECs were incubated with endotoxin free rAtl (20 μg/mL) for the indicated times, and cell lysates were subjected to Western blot to assess the expression of phospho-MLC and MLC. Band intensity relative to the uninfected group was analyzed. (D) Effect of the MLCK inhibitor, ML-7, on SS2 penetration in the tracheal barrier model. STEC monolayers on Transwell inserts were pretreated with DMSO or ML-7 for 1 h before infection with WT SS2 or with the Δ*atl* mutant strain (MOI 50:1), and the penetration rates of SS2 from the apical side of Transwell inserts to the basolateral side were calculated at 3 hpi. (E) STECs pretreated with DMSO or ML-7 for 1 h were infected with WT SS2 or with the Δ*atl* mutant strain (MOI 50:1), and the expression of occludin in the cell membrane was determined by Western blot at 3 hpi. Band intensity relative to the control was analyzed. (F) Immunofluorescence staining was used to analyze the effect of ML-7 on the distribution of occludin in STECs. STECs pretreated with DMSO or ML-7 for 1 h were infected with WT SS2 or with the Δ*atl* mutant strain (MOI 50:1), and the distribution of occludin (green) in STECs was then determined by immunofluorescence staining. The cell nuclei were stained with DAPI (blue). Scale bar, 50 μm. The data shown are representative or are presented as the mean ± SD of the values obtained in three independent experiments. Statistical analysis was performed using one-way ANOVA with Dunnett’s multiple comparison test (A-C) or Tukey’s multiple comparison test (D-E). ns, not significant; *, *P* < 0.05; **, *P* < 0.01; ***, *P* < 0.001; ****, *P* < 0.0001.

Actin and myosin regulate paracellular permeability through MLC phosphorylation [25, 26]. Phosphorylation of MLC in response to multiple types of stimuli has been reported to increase the permeability of the epithelial barrier [27–29]. Western blot was used to assess the effect of SS2 on the phosphorylation of MLC. As shown in Fig 3A, SS2 infection resulted in a time-dependent increase in the phosphorylation of MLC, and this phosphorylation was significantly lower in the Δ*atl* infection group than in the WT infection group (Fig 3B). Moreover, purified rAtl increased the phosphorylation of MLC in STECs in a time-dependent manner (Fig 3C).

Subsequent experiments showed that after pretreatment of STEC monolayers on Transwell inserts with the MLCK pathway inhibitor ML-7, there were significantly fewer CFUs in the WT infection group than in the control group, while the penetration competency of Δ*atl* was not affected (Fig 3D). ML-7 prevented the decrease in TEER values that was otherwise observed when STEC monolayers were challenged with WT SS2 (S9 Fig) and significantly prevent the the translocation of occludin from the cell membrane into the cytoplasm induced by WT SS2 (Fig 3E). Consistent with the Western blot results, immunofluorescence staining with antibodies against occludin indicated that ML-7 hindered the appearance of granular occludin in the cytoplasm and facilitated the continuous distribution of occludin in the cell membranes of STECs (Fig 3F). These results demonstrate that Atl promotes MLC phosphorylation and thereby increases tracheal epithelial permeability.

### Atl interacts with host vimentin as an adhesin

**Fig 4:**
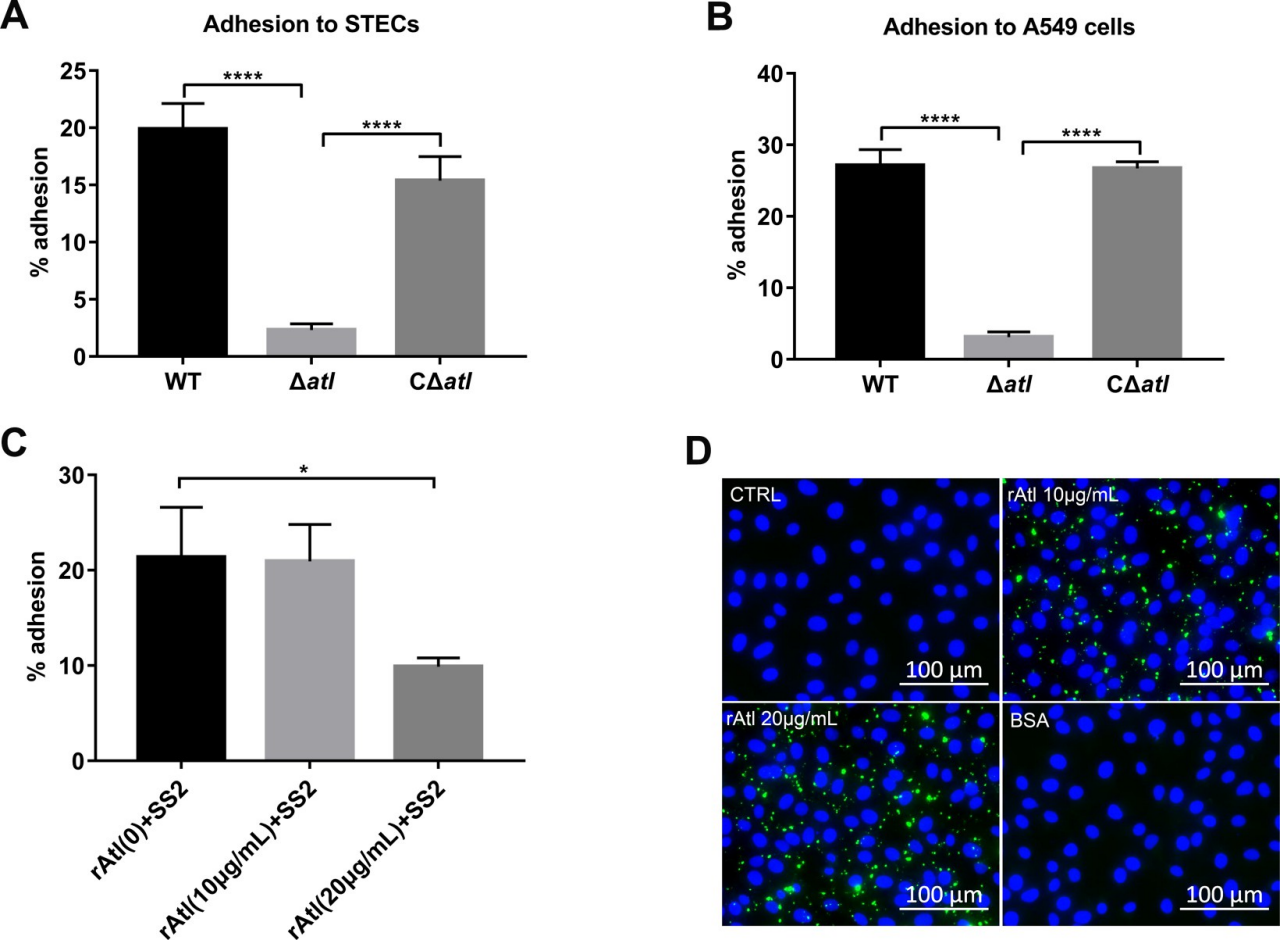
Atl is a main adhesin in SS2. (A and B) Analysis of the adhesion of WT, Δ*atl*, and CΔ*atl* to STECs (A) and A549 cells (B) after a 2 h infection. (C) Effect of rAtl on the adhesion of SS2. Untreated STECs and STECs that had been pretreated with the indicated concentrations of rAtl were infected with WT SS2 for 2 h, and the adherence of WT SS2 to the STECs was then assessed. (D) STECs were incubated with the indicated concentrations of rAtl, and the adherence of rAtl (green) to the cell surface was assessed by immunofluorescence staining. STECs that had been incubated with BSA were used as a negative control. The cell nuclei were stained with DAPI (blue). Scale bar, 100 μm. The data shown are representative or are presented as the mean ± SD of the values obtained in three independent experiments. Statistical analysis was performed using one-way ANOVA with Dunnett’s multiple comparison test (A-C). *, *P* < 0.05; ****, *P* < 0.0001.

Analysis of the rate of adhesion of WT, Δ*atl*, and CΔ*atl* to A549 cells and STECs indicated that the adhesion of Δ*atl* mutant to STECs was 10 times lower than that of WT SS2 (Fig 4A); similar results were obtained in A549 cells (Fig 4B). In contrast, the adhesion rate of CΔ*atl* to STECs and A549 cells was significantly higher than that of Δ*atl* mutant (Fig 4A and 4B). To further demonstrate the role of Atl in adhesion, the effect of added rAtl protein on SS2 adhesion was determined. As shown in Fig 4C, preincubation of STECs with rAtl resulted in a significant decrease in the adhesion rate. In particular, preincubation with 20 μg/mL rAtl reduced SS2 adhesion by 50%. Indirect immunofluorescence staining showed that rAtl adhered to the surface of STECs (Fig 4D). These results indicate that Atl acts as an adhesin of SS2 that mediates adhesion to STECs.

**Fig 5:**
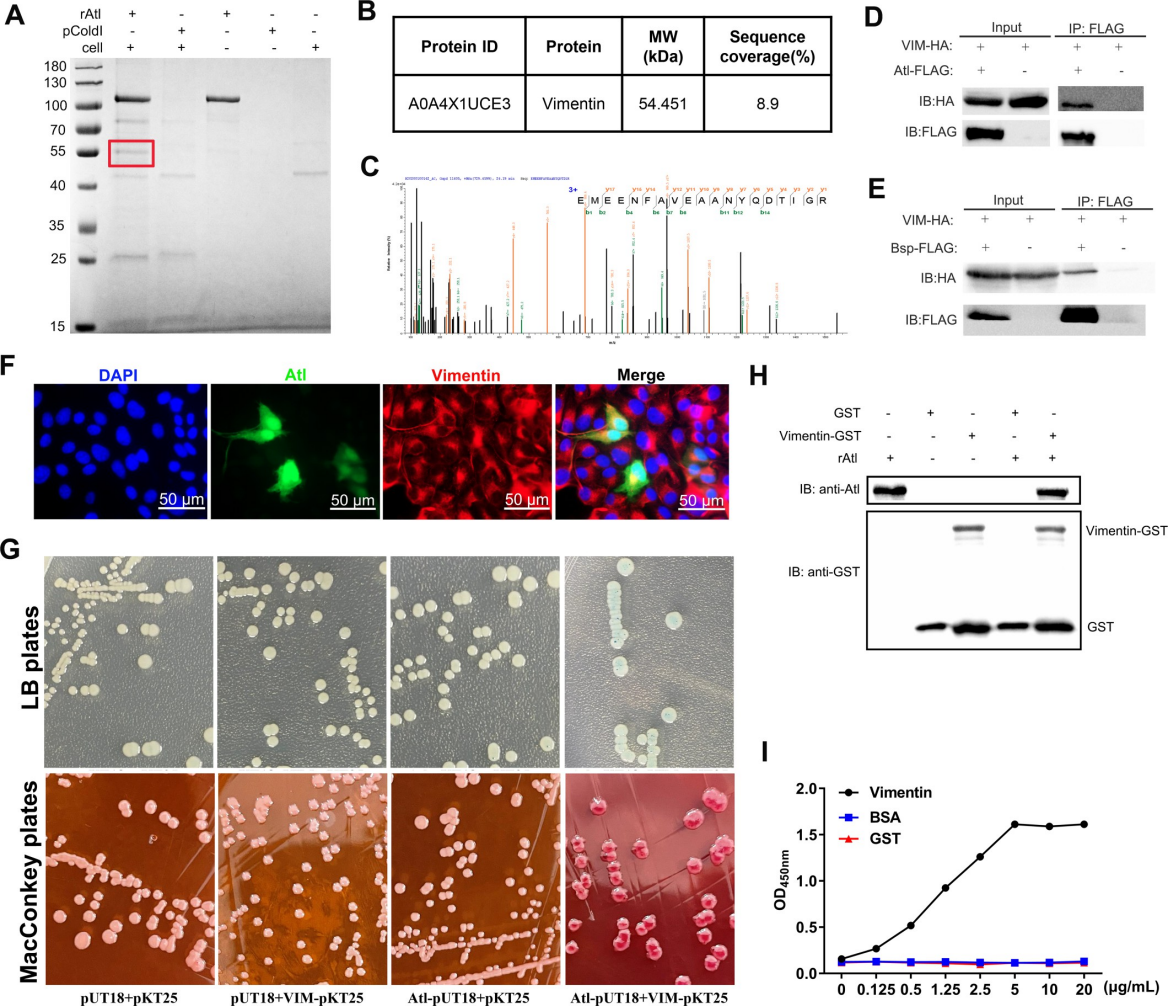
Atl directly interacts with host vimentin. (A) Pull-down assay of proteins that interact with Atl in STECs. STEC lysates were incubated with His-tagged Atl for 2 h. The final eluted proteins were analyzed by Coomassie Brilliant Blue. The protein bands (red frame) in the experimental lanes that were not present in the control lanes were excised and subjected to LC-MS/MS. (B) LC-MS/MS analysis result for vimentin. (C) LC-MS/MS spectrum for the vimentin peptide. (D) Co-IP of FLAG-tagged Atl and HA-tagged vimentin. The input is the immunoblot of Atl and vimentin in cell lysates. The output is the immunoblot of Atl and vimentin in the eluate after co-IP. (E) Co-IP analysis of the interaction between the Bsp domain of Atl and vimentin. The input is the immunoblot of Bsp domain and vimentin in cell lysates. The output is the immunoblot of Bsp domain and vimentin in the eluate after co-IP. (F) Immunofluorescence analysis showing the colocalization of GFP-tagged Atl (green) and endogenous vimentin (red) in STECs transfected with Atl-pAcGFP1-C plasmid. The cell nuclei were stained with DAPI (blue). Scale bar, 50 μm. (G) Bacterial two-hybrid assay to detect the interaction between Atl and vimentin. Blue colonies formation on LB plates containing X-Gal and red colonies formation on MacConkey plates containing maltose suggest that a direct interaction occurs. (H) GST pull-down assay to determined the interaction between Atl and vimentin. GST or vimentin-GST expressed in *E*. *coli* Rosetta (DE3) strains were conjugated to glutathione beads and incubated with His-tagged Atl. After being washed, the bound protein complex were analyzed by immunoblotting with anti-GST mAb and anti-Atl polyclonal antibody. (I) Saturation binding experiments with increasing amount of vimentin-GST (black line), GST (red line) and BSA (blue line) (0.1 to 20 μg/mL). Anti-Atl polyclonal antibody against the Atl protein and HRP-conjugated goat anti-rabbit IgG antibody were added as primary and secondary antibody, respectively, and the binding was detected by ELISA at 450 nm. The data shown are representative or are presented as the mean ± SD of the values obtained in three independent experiments.

Given the role of Atl in adhesion, we speculated that Atl mediates adhesion to STECs by binding to STEC surface receptors. A pull-down assay performed after incubation of STEC cell lysates with His-tagged rAtl yielded a protein band at approximately 55 kDa (Fig 5A). The protein band was extracted from the polyacrylamide gel and subjected to liquid chromatography-tandem mass spectrometry (LC-MS/MS) analysis (Fig 5B and 5C). To confirm the interaction of Atl with the candidate host protein, co-immunoprecipitation (co-IP) was conducted using lysates of HEK293T cells co-transfected with FLAG-tagged Atl and HA-tagged candidate interaction protein. As shown in Fig 5D, HA-tagged vimentin co-immunoprecipitated with FLAG-tagged Atl. Considering that Atl harbors MurNAc-LAA and the group B *Streptococcus* (GBS) Bsp-like domain in SS2, we next explored the domain that interacts with vimentin. As shown in Fig 5E, HA-tagged vimentin co-immunoprecipitated with FLAG-tagged Bsp. Immunofluorescence showed that overexpressed GFP-tagged Atl colocalized with vimentin in STECs (Fig 5F). This interaction was further confirmed in a bacterial adenylate cyclase-based two-hybrid (BACTH) system. After transformation with the Atl-pUT18 and vimentin-pKT25 plasmids, the *E*. *coli* strain BTH101 lacking adenylate cyclase (*cyaA*) formed blue colonies when grown on LB plates containing X-Gal and red colonies when grown on MacConkey plates containing maltose, indicating the presence of β-galactosidase activity, while the negative control fromed white colonies (Fig 5G). To investigate the possibility that a direct association between Atl and vimentin occurs, we performed an *in vitro* GST pull-down assay using rAtl and GST-tagged vimentin (vimentin-GST). As shown in Fig 5H, rAtl was pulled down with vimentin-GST. Furthermore, a saturation binding experiment in which microplate wells coated with increasing amounts of vimentin-GST were challenged with 20 μg/mL rAtl revealed that vimentin-GST readily bound to rAtl in a concentration-dependent manner (Fig 5I). When the microplates were coated with the indicated concentrations of GST and BSA, no binding occurred in the presence of rAtl (Fig 5I). Taken together, these observations suggest that the GBS Bsp-like domain of Atl binds directly to vimentin on the surface of STECs.

### Vimentin is necessary for MLCK-mediated penetration of the tracheal epithelial barrier

**Fig 6:**
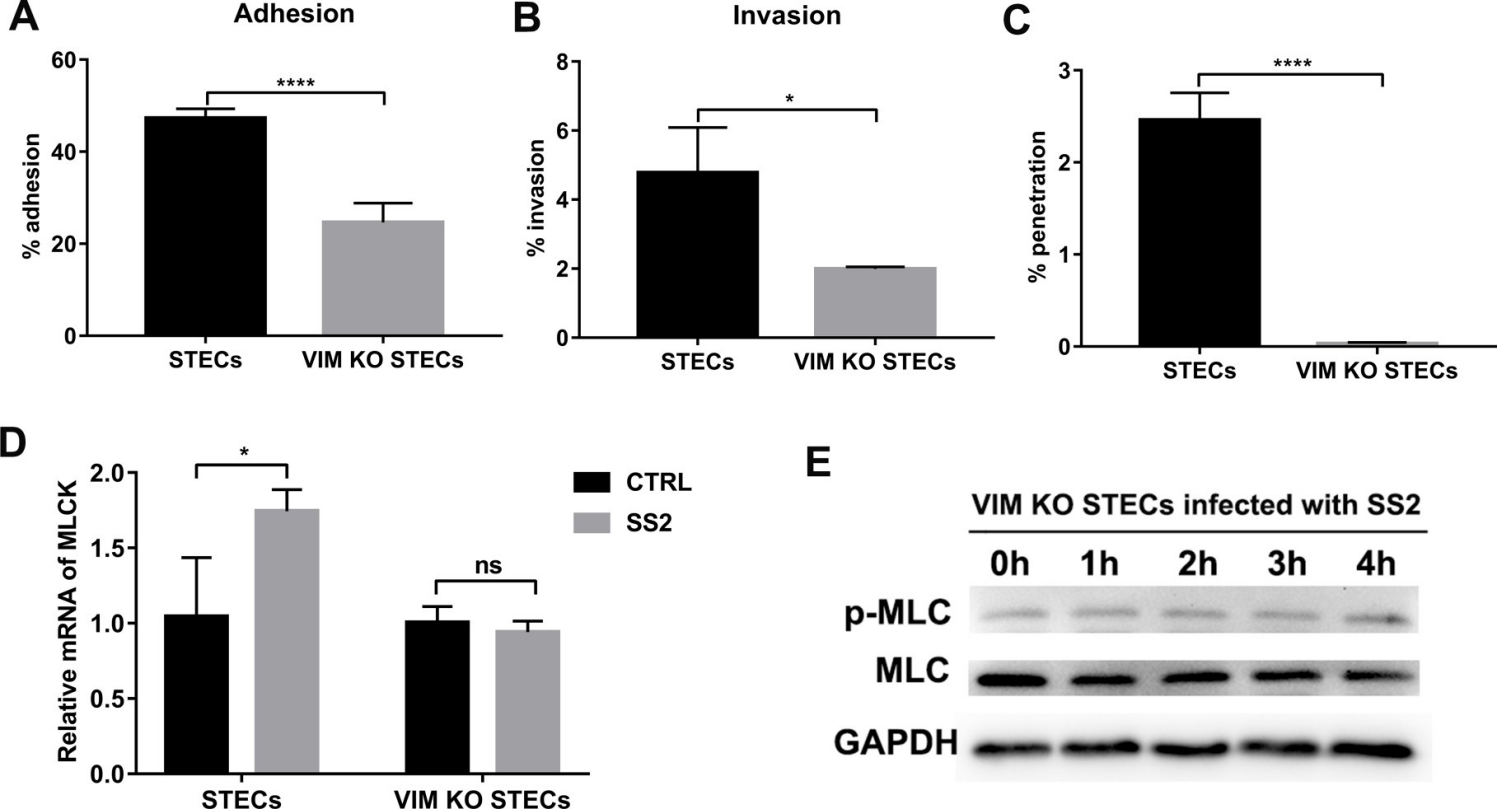
Vimentin is required for MLCK-mediated penetration of the respiratory barrier. (A and B) Analysis of adhesion (A) and invasion (B) of WT SS2 to STECs or VIM KO STECs after a 2 h infection. (C) Determination of the ability of WT SS2 to penetrate VIM KO STEC and STEC monolayer on Transwell inserts at 3 hpi. (D) Effect of SS2 infection on the transcription of MLCK in STECs and VIM KO STECs. STECs and VIM KO STECs were infected with WT SS2 for 3 h, and total RNA was isolated and subjected to qRT-PCR to analyze the transcription of MLCK. (E) Effect of SS2 infection on the phosphorylation of MLC in VIM KO STECs. VIM KO STECs were infected with WT SS2 (MOI 50:1) for the indicated times, and cell lysates were prepared and subjected to Western blot to assess the expression of phospho-MLC and MLC. The data shown are representative or are presented as the mean ± SD of the values obtained in three independent experiments. Statistical analysis was performed using the unpaired t test (A-C) and two-way ANOVA followed by Bonferroni’s multiple comparisons test (D). ns, not significant; *, *P* < 0.05; ****, *P* < 0.0001.

To explore the role of vimentin in infection of the tracheal epithelium by SS2, lentiviruses containing the single-guide RNA (sgRNA)-CRISPR/Cas9 plasmid targeting the vimentin gene were used to infect STECs. The qRT-PCR and Western blot results showed that the on-target efficiency of the three sgRNAs reached 80% at 36 hpi (S10A–S10C Fig). A vimentin knockout STEC cell line (VIM KO STEC) was generated using puromycin screening and verified by Western blot and immunostaining (S10D and S10E Fig). The adhesion and invasion rates of SS2 to VIM KO STECs were more than 2-fold lower than those observed in the control group (Fig 6A and 6B). Notably, the ability of SS2 to penetrate VIM KO STEC monolayers on Transwell inserts was significantly reduced (Fig 6C). These results suggest that vimentin is required for SS2 infection through the tracheal epithelium.

Next, the effects of vimentin on the transcription of MLCK and the phosphorylation of MLC were measured by qRT-PCR and Western blot. Intriguingly, the results indicated that SS2 infection significantly increased the transcription of MLCK in STECs but had no effect on the transcription of MLCK in VIM KO STECs (Fig 6D). In addition, SS2 infection significantly increased the phosphorylation of MLC in STECs (Fig 3A), but did not affect the phosphorylation of MLC in VIM KO STECs (Fig 6E). Overall, these observations demonstrate that vimentin promotes MLCK-mediated opening of the epithelial barrier.

### Vimentin contributes to SS2 penetration of the tracheal epithelium and systemic dissemination *in vivo*

**Fig 7:**
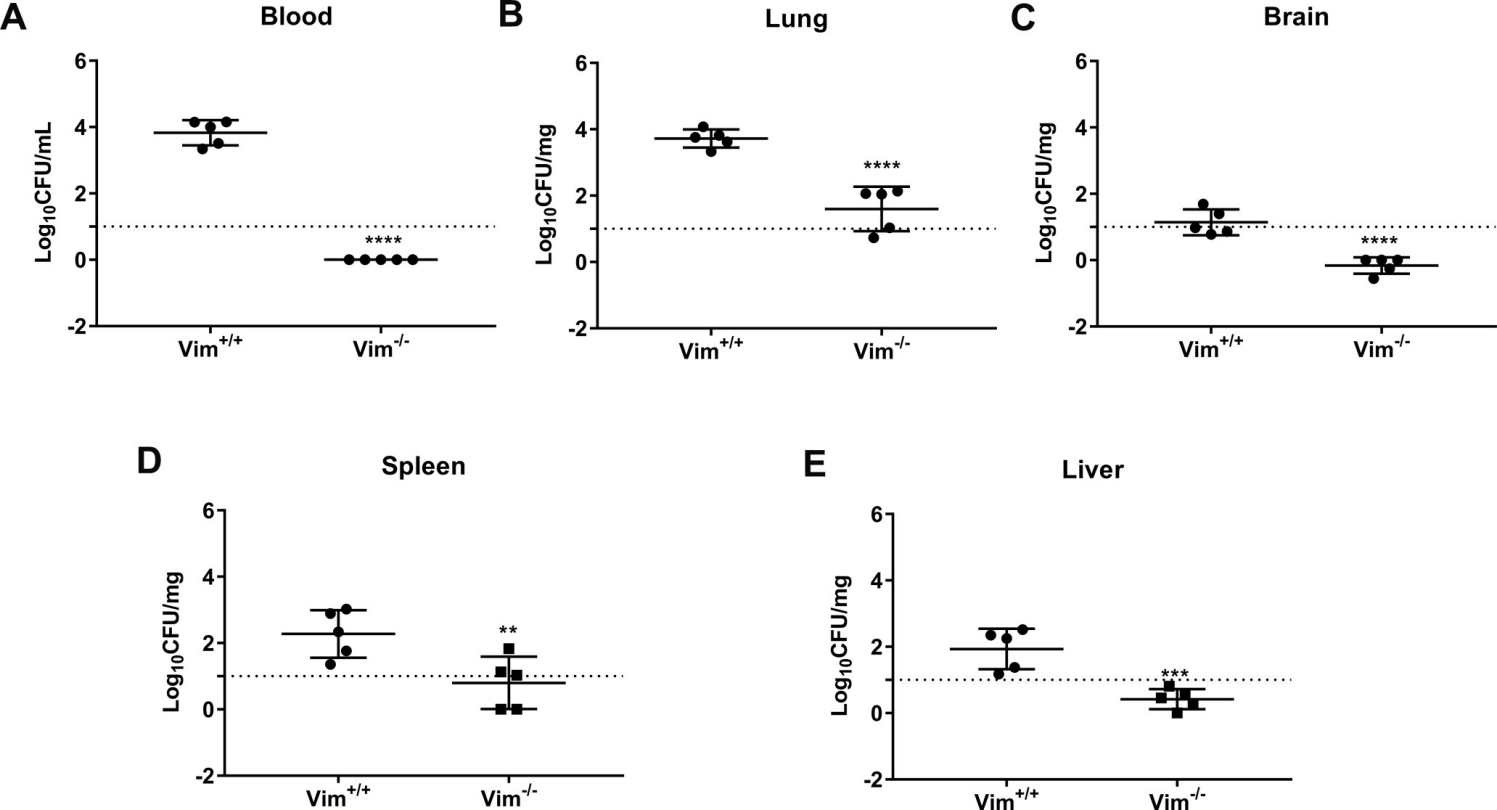
Vimentin contributes to SS2 penetration of the tracheal epithelium *in vivo*. (A-E) Vim^+/+^ and Vim^-/-^ mice were intranasally inoculated with 1 × 10^9^ CFUs WT SS2, and the bacterial loads in the blood (A), lung (B), brain (C), spleen (D) and liver (E) were determined at 24 hpi (n = 5 mice/group). The data are from representative experiments and are presented as the mean ± SD. Statistical analysis was performed using the unpaired t test (A-E). **, *P* < 0.01; ***, *P* < 0.001; ****, *P* < 0.0001.

To further verify the role of vimentin in respiratory tract infection caused by SS2, vimentin knockout C57/BL6J (Vim^-/-^) mice were obtained. And we replicated the experimental results of the BALB/c mouse model in Fig 1 using the C57/BL6J mouse model (Figs 7 and S11). Wild type C57/BL6J (Vim^+/+^) and Vim^-/-^ mice were administered the WT or Δ*atl* strain intranasally, and the bacterial loads in the blood, lung, brain, liver and spleen at 24 hpi were determined. The results showed that fewer WT bacteria were recovered from the tissues of Vim^-/-^ mice than from the tissues of Vim^+/+^ mice (Fig 7). However, Vim^+/+^ and Vim^-/-^ mice infected with the Δ*atl* mutant showed no significant difference in tissue bacterial load (S11 Fig). Together, these data suggest that vimentin is indispensable for SS2 translocation across the tracheal epithelium and for the occurrence of invasive infection.

## Discussion

Intranasal infection models in C57BL/6J and BALB/c mice and a Transwell tracheal epithelial barrier model were constructed in this study. Screening of the TnYLB-1 insertion mutant library of ZY05719 indicated that Atl plays a critical role in penetration of the tracheal epithelial barrier by SS2. Deletion of *atl* markedly diminished the capacity of SS2 to penetrate the tracheal epithelium and STEC monolayers in Transwell inserts. Further experiments showed that Atl binds directly to vimentin on the surface of STECs, and knockout of vimentin in STEC monolayers resulted in reduced bacterial penetration and reduced MLC phosphorylation. In addition, vimentin null mice infected with SS2 exhibited decreased bacterial counts in tissues and tracheal and lung damage. Our novel findings provide evidence that by interacting with vimentin, Atl promotes MLCK-mediated increase in the permeability of the tracheal epithelium and facilitates SS2 penetration.

In normal-growing bacteria, bacterial autolysin is mainly involved in the hydrolysis of bacterial peptidoglycan, and the synthesis of autolysin is strictly regulated [30]. Bacterial autolysin also plays diverse roles in virulence. For example, in *Staphylococcus aureus*, autolysin modulates the production of extracellular membrane vesicles (EVs) by altering the permeability of the cell wall, thereby prompting bacterial pathogenicity [31]. Increased LytA in *Streptococcus pneumoniae* contributes to the release of more pneumolysin, resulting in destruction of the epithelial barrier and dissemination of bacteria into the bloodstream [32]. In this study, Atl, as a key pathogenic factor of SS2, contributed to penetration of the respiratory barrier in both C57BL/6J and BALB/c mouse models. This finding is supported by the fact that rAtl protein alone increased the permeability of STEC monolayers. Notably, Atl-mediated penetration of the epithelial barrier was not due to cellular cytotoxicity but instead to redistribution of TJs through MLCK activation, since activated MLCK induces assembly and disassembly of TJs [17]. This finding is further supported by our observations that rAtl increased the phosphorylation of MLC and that pharmacological inhibition of MLC phosphorylation inhibited bacterial penetration and membrane TJ destruction.

As the structural basis for maintaining the epithelial barrier, the distribution of TJs in epithelial cell membrane and cytoplasm is highly dynamic and influenced by various factors in the environment [11, 33]. In this study, SS2 infection did not cause degradation or decreased synthesis of TJ proteins in STECs; instead, it caused translocation of occludin and claudin1 from the cell membrane to the cytoplasm, resulting in a decrease in the distribution of TJ proteins in the cell membrane and an increase in barrier permeability. Immunofluorescence confirmed that after infection with WT SS2, ZO-1 and occludin exhibited a discontinuous distribution in the cell membrane and occludin exhibited a granular distribution in the cytoplasm. Pathogens traverse the epithelial barrier by changing the distribution of TJs and this has been demonstrated in a number of studies. For example, listeria adhesion protein (LAP) redistributes intestinal epithelial TJ proteins (claudin1 and occludin) and AJ proteins (E-cadherin), resulting in increased intestinal barrier permeability, when *Listeria monocytogenes* penetrates the intestinal tract [34]. Enteropathogenic *Escherichia coli* (EPEC) infection can cause endocytosis of occludin in the cell membrane, resulting in increased intestinal barrier permeability [35]. TJ proteins are known to internalize via caveolin-1-dependent endocytosis, micropinocytosis, and clathrin-mediated endocytosis [36, 37]. The precise mechanism of occludin redistribution caused by SS2 infection requires further exploration.

We observed that adhesion of SS2 was significantly reduced after *atl* gene deletion and that rAtl significantly inhibited SS2 adhesion. Our observation supports the idea that Atl plays a crucial role in initiating infection as an adhesin [38]. There have been reports of identified receptors that bind to autolysin on the cell surface. *Streptococcus mutans* autolysin AtlA can bind to fibronectin [39]. The autolysin/adhesin Atl and AtlE of *S*. *aureus* and *Staphylococcus epidermidis*, respectively, can bind to the heat shock homologous protein Hsc70 and thereby contribute to internalization [38]. In this study, we found that Atl interacts directly with a specific cellular receptor, vimentin. The interaction of Atl with vimentin led to a leakier barrier, which was conducive to SS2 penetration through the paracellular pathway. The role of other host factors that can bind to Atl remains to be further explored, as these findings may enhance our understanding of the diverse roles played by Atl in bacterial virulence.

Vimentin is a type III intermediate filament (IF) protein involved in various functions, including maintenance of cytoskeletal integrity, cell division, intracellular signal transduction, cell adhesion, and cell migration [40–42]. Accumulating evidence supports the idea that vimentin plays diverse role in bacterial infections. In mouse brain microvascular endothelial cells, vimentin serves as the receptor for the *L*. *monocytogenes* surface protein InlF, and its interaction with InlF is critical for colonization of the brain by *L*. *monocytogenes* [43]. IbeA of *E*. *coli* K1 interacts with vimentin in human brain microvascular endothelial cells (HBMECs), facilitating invasion [44]. The interaction between vimentin and BspC of GBS promotes adhesion to BMECs and the development of meningitis [45]. In this study, vimentin was found to be required for SS2 infection, as vimentin null mice showed less susceptibility to SS2 infection and the ability of SS2 to penetrate the vimentin knockout STEC monolayers was significantly impaired. In addition, unlike the time-dependent increase in MLC phosphorylation observed in SS2-infected STECs, SS2 infection had no effect on MLC phosphorylation levels in VIM KO STECs. Our results support the idea that vimentin is associated with signal transduction [46–48] and further show that the Atl-vimentin interaction is required for the phosphorylation of MLC. Further work will be required to elucidate the precise mechanisms by which vimentin affects MLC phosphorylation.

**Fig 8:**
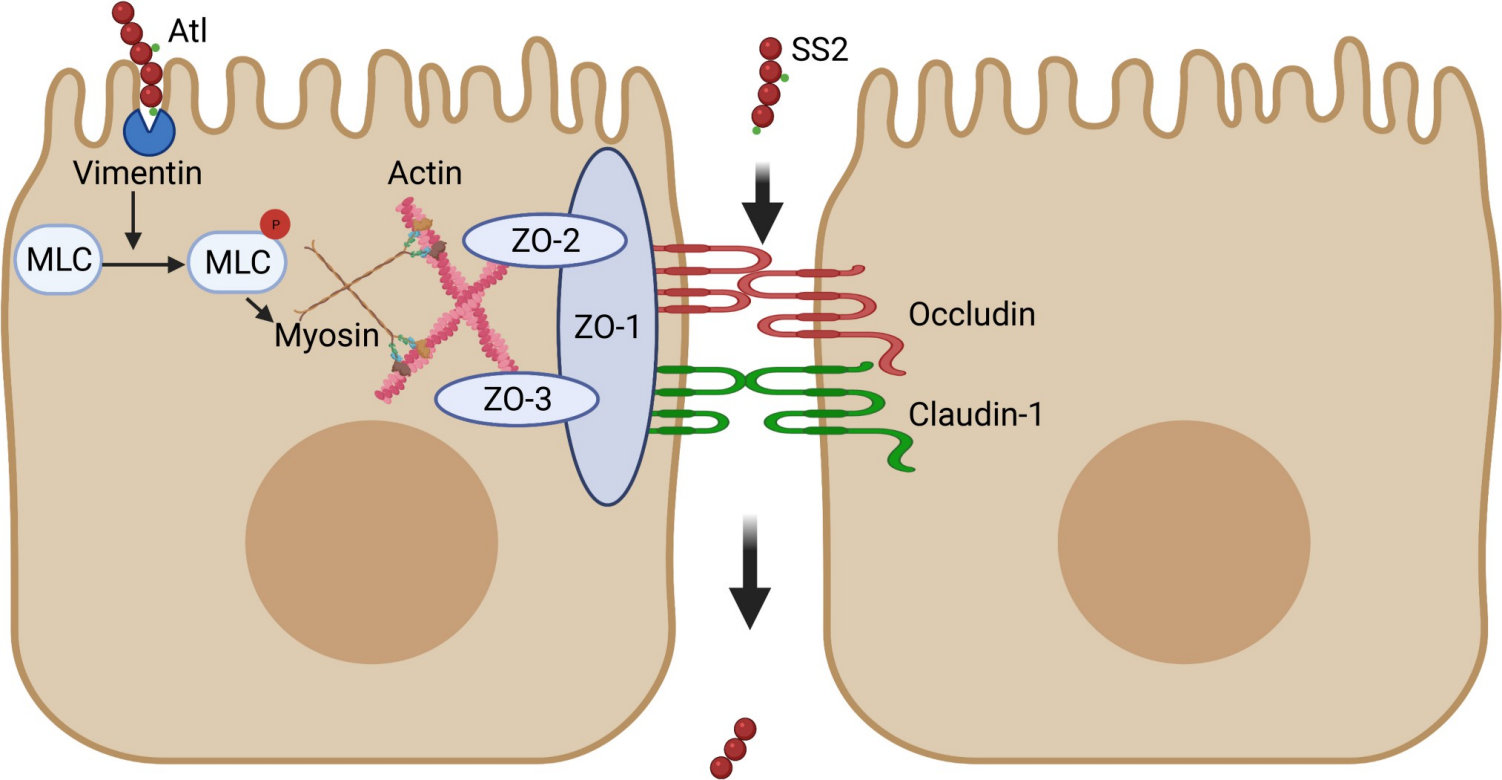
Schematic summary of the process through which SS2 penetrates the tracheal epithelial barrier. Atl first promotes the adhesion of SS2 to the STEC surface. Then, Atl interacts with vimentin on the surface of STECs. The reassembly of vimentin activates MLCK signaling. Subsequently, phosphorylated MLC induces the redistribution of TJs and disruption of the tracheal epithelial barrier. Finally, SS2 penetrates the barrier from the paracellular pathway.

The respiratory tract is the primary entry site for various pathogens, and respiratory tract infections are a leading cause of morbidity and mortality in children and the elderly worldwide [49, 50]. Diverse bacterial species, including *S*. *suis*, that colonize mucosal surfaces are occasionally able to breach the epithelium and cause invasive disease. However, how these respiratory pathogens penetrate the respiratory barrier remains unclear. This study demonstrates a novel mechanism of SS2 infection through the respiratory tract; that is, Atl promotes MLCK-mediated opening of the tracheal epithelial barrier by binding to vimentin on the surface of STECs (Fig 8). SS2 Atl harbors the GBS Bsp-like domain and the MurNAc-LAA domain. Here, we found that the GBS Bsp-like domain of Atl binds to vimentin. Comparison of Atl protein sequences using the Basic Local Alignment Search Tool (BLAST) provided by the National Center for Biotechnology Information (NCBI) indicated that the GBS Bsp-like domains of respiratory pathogens, such as *S*. *pneumoniae*, *Klebsiella pneumoniae*, and *Streptococcus pyogenes*, have high homology (up to 60%) with the GBS Bsp-like domain of Atl (S12 Fig). Therefore, whether the GBS Bsp-like domains of these respiratory pathogens play the same role in the process of breaching the tracheal epithelial barrier and systemic dissemination requires further investigation. This study elucidated the mechanisms by which SS2 breaches the respiratory barrier to cause systemic infection and may serve as a reference for future studies on other respiratory pathogens. Moreover, Atl’s capacity to increase tracheal epithelial barrier permeability may provide potential medical applications in drug delivery and vaccine design.

## Materials and methods

### Ethics statement

All animal experiments were approved by the Laboratory Animal Welfare and Ethics Committee of Nanjing Agricultural University, China (PZB2020017, NJAU.No20220306028), and conducted in accordance with animal welfare standards. All efforts were made to minimize the animals’ suffering.

### Bacterial strains and plasmids

The WT SS2 strain ZY05719 was isolated from dead pigs during an outbreak of streptococcal disease in Sichuan Province, China, in 2005. SS2 was grown in Todd-Hewitt Broth (THB, Becton, Franklin Lakes, NJ, USA) containing 2% yeast extract (Oxoid Ltd., UK) (THY). All SS2 strains were grown to mid-log phase (an optical density at 600 nm (OD_600_) of 0.4–0.8) at 37°C, washed three times with PBS and resuspended in DMEM unless otherwise indicated. *E*. *coli* was grown in Luria broth (LB, Oxoid Ltd.) or in LB supplemented with 1.5% agar. When necessary, spectinomycin (Spc, *E*. *coli* 50 μg/mL, SS2 100 μg/mL), kanamycin (Kan, 50 μg/mL), ampicillin (Amp, 100 μg/mL), or chloramphenicol (Cm, 34 μg/mL) was added to the culture medium.

For the expression of His- or GST-tagged proteins, *atl* devoid of the signal peptide-encoding sequence was cloned into the *Bam*H I and *Hin*d III sites of pCold-I, and the *vimentin* gene was amplified from swine cDNA and subcloned into pGEX-6p-1. *E*. *coli* BL21 (DE3) and *E*. *coli* Rosetta (DE3) were used to express Atl and vimentin, respectively. For the expression of proteins in eukaryotic cells, *atl* devoid of the signal peptide-encoding sequence was cloned into pcDNA3-2×FLAG and pAcGFP1-C; the *vimentin* gene was cloned into pCAGGS. For the expression of proteins in *E*. *coli* lacking *cyaA*, *atl* devoid of the signal peptide-encoding sequence was cloned into pUT18, and the *vimentin* gene was cloned into pKT25. All plasmid constructs were verified by Sanger sequencing and propagated in *E*. *coli* strain DH5α. The primers used in this study are listed in S2 Table.

### Cell lines and culture conditions

STECs, A549 cells, and HEK293T cells (ATCC source) were cultured in an incubator containing 5% CO_2_ at 37°C. The cells were cultured in high glucose Dulbecco’s modified Eagle’s medium (DMEM, Gibco, Grand Island, NY, USA) supplemented with 10% heat-inactivated fetal bovine serum (FBS, Gibco).

To construct a tracheal epithelial barrier model *in vitro*, STECs were seeded on Transwell inserts with a 3.0-μm-pore PET membrane (Corning, NY, USA) at a density of 5×10^4^ cells/cm^2^, and the culture medium was changed every 2 d. A Millicell-ERSII A voltmeter (Millipore, MA, USA) was used to measure transepithelial electrical resistance (TEER).

### Δ*atl* and complementary strain construction

The Δ*atl* mutant was generated in ZY05719 by homologous recombination using a previously described method [51]. The fragments upstream and downstream of the *atl* gene were amplified from the ZY05719 genome using the primers Δ*atl* -F1/Δ*atl* -R1 and Δ*atl* -F2/Δ*atl* -R2. Then, the fusion fragment was obtained using the primer pair Δ*atl* -F1/Δ*atl* -R2, using the PCR products of those two flanks as a template and cloned into the *Eco*R I and *Sal* I restriction sites of the temperature-sensitive *S*. *suis*-*E*. *coli* shuttle vector pSET4s using the ClonExpress II One Step Cloning Kit (Vazyme Biotech Co., China). The Δ*atl*-pSET4S plasmid was electroporated into ZY05719 using the Gene Pulser XCell electroporation system (Bio-Rad, Hercules, CA, USA) (voltage: 2500 V, capacitance: 25 μF, resistance: 200 Ω, cuvette: 1 mm), and mutant isolation was conducted as described [51]. The complementary strain of the Δ*atl* mutant was constructed using the same in-frame allelic replacement method; there was a nonsense mutation at the 3096 nucleotide to distinguish the WT strain. The primers used to construct the bacterial strains are listed in S2 Table.

### Animal experiments

Four-week-old female BALB/c mice were purchased from the Comparative Medicine Center of Yangzhou University (Yangzhou, China), and four to five-week-old wild type C57BL/6J (Vim^+/+^) and vimentin knockout (Vim^-/-^) mice were purchased from GemPharmatech (Nanjing, China). All mice were housed in the Barrier Environment at the Laboratory Animal Center of Nanjing Agricultural University. To compare the abilities of WT and Δ*atl* to penetrate the tracheal barrier in mice, 21 BALB/c mice, 15 Vim^+/+^ mice or 15 Vim^-/-^ mice were randomly divided into three groups, and the mice were intranasally inoculated with PBS, WT, or the Δ*atl* mutant strain (1 × 10^9^ CFUs). The blood and organ homogenates were diluted and spread on THY agar plates to determine the bacterial load in the blood and other organs. For histopathological analysis, the trachea and lungs of infected BALB/c mice were fixed and embedded in paraffin to create paraffin blocks, and the blocks were then sectioned and subjected to hematoxylin and eosin (H&E) staining or immunofluorescence. Microscopic lesions, including alveolar and interstitial edema, hemorrhage, inflammatory cell infiltration, tracheal epithelial ciliary disruption, and epithelial cell translocation, were evaluated in from five random fields of each tissue section in a blinded manner and scored as follows: normal = 0, mild = 1, moderate = 2, severe = 3 and very severe = 4.

### Protein expression and purification

The recombinant *E*. *coli* BL21 (DE3) strain carrying plasmid *atl*-pCold-I was cultured in LB containing 100 μg/mL Amp, and the *E*. *coli* Rosetta (DE3) strain carrying plasmid *vimentin*-pGEX-6p-1 was cultured in LB containing 100 μg/mL Amp and 34 μg/mL Cm. When the OD_600_ of the culture reached 0.8–1.0, the protein was induced by the addition of isopropyl-β-d-1-thiogalactopyranoside (IPTG, Sigma-Aldrich, St. Louis, MO, USA) to the culture to a concentration of 0.2 mM and incubation at 16°C with shaking (90 rpm) for 18 h.

To purify His-tagged Atl, bacteria were collected by centrifugation and resuspended in binding buffer (20 mM Na_3_PO_4_, 20 mM imidazole, 0.5 M NaCl, pH 8.0) containing 1 mM phenylmethylsulfonyl fluoride (PMSF) for ultrasonic purification. HisTrap HP (5 mL; GE Healthcare, Piscataway, NJ, USA) was used to purify rAtl. Then the endotoxin was removed from purified rAtl using high capacity endotoxin removal resin (Pierce, Rockford, IL, USA).

To purify GST-tagged vimentin (vimentin-GST) and GST protein, bacteria were collected and resuspended in PBS containing 1 mM PMSF. After ultrasonication, vimentin-GST and GST protein were purified using GST-tag purification resin (Beyotime, Shanghai, China).

### Determination of epithelial permeability and bacterial penetration

STEC monolayers in Transwell inserts were incubated with endotoxin free rAtl for 12 h, 24 h, and 36 h, washed with Hank’s balanced salt solution (HBSS, Sangon Biotech, Shanghai, China), and then incubated with DMEM containing 1 mg/mL 4 kDa FITC-dextran (FD-4, Sigma-Aldrich) for 1 h. The FD-4 flux into the basolateral chamber was evaluated in a SPARK multimode microplate reader (TECAN, Switzerland) using 485-nm excitation and 535-nm emission filters. The penetration rate of FD-4 in the control group was set as 1%, and the penetration rate of FD-4 in other groups was compared with that in the control group.

To analyze the ability of bacteria to penetrate the tracheal barrier model, confluent STEC monolayers on Transwell inserts were infected with SS2 at a multiplicity of infection (MOI) of 50:1. After serial dilution following incubation at 37°C in a 5% CO_2_ incubator for the indicated times, 100 μL of the medium was removed from the basolateral side of the Transwell insert and spread on agar plates to enumerate the CFUs. The penetration rates were determined by calculating the ratio of CFUs in the basolateral medium to CFUs in the apical side of the Transwell insert.

### Adhesion assay

The adhesion ability of SS2 to STECs was analyzed as described previously (32) with slight modifications. Briefly, confluent STEC monolayers in 24-well plates were infected with SS2 strains at an MOI of 50:1. To determine the influence of rAtl on adhesion, STEC monolayers were not treated with rAtl or were pretreated with endotoxin free rAtl for 1 h before infection with WT SS2. Two hours after infection, the cells were lysed in 1 mL of sterile water, and the lysates were spread on THY agar plates for CFU determination. Three replicate wells were used in each experiment, and the experiment was repeated three times.

### Analysis of the effect of SS2 on TJ proteins in STECs

SS2 was used to infect STECs in six-well plates at an MOI of 50:1, followed by incubation at 37°C in a 5% CO_2_ incubator. For the detection of TJ protein levels, 1 mL of RIPR (1 mM PMSF was added before use) was added to the cultures, and the lysates were centrifuged at 12,000 × *g* for 10 min at 4°C to obtain the supernatant. A membrane and cytosolic protein extraction kit (KeyGEN Biotech, Nanjing, China) was used to extract the cell membrane and cytoplasmic proteins. The extracted proteins were then subjected to SDS-PAGE.

### Quantitative RT-PCR

STECs were lysed with TRIzol (Vazyme Biotech Co., China) and total RNA was isolated according to the protocol provided by the manufacturer. cDNA was synthesized using HiScript Q RT SuperMix for qPCR (+gDNA wiper) (Vazyme Biotech Co.). qRT-PCR was performed on a 7300 Real-Time PCR System (Applied Biosystems, Foster City, California, USA) with ChamQ Universal SYBR qPCR Master Mix (Vazyme Biotech Co.). The primer sequences are shown in S2 Table. The *GAPDH* gene was used as an internal control, and relative quantification compared to the uninfected cells was calculated using the 2^−ΔΔCt^ method [52]. The tests were performed in triplicate and each set of qPCR assays was repeated three times.

### Western blots

Electrophoretically separated proteins were transferred to polyvinylidene fluoride (PVDF) membranes (Millipore, USA), which were then blocked with 3% bovine serum albumin (BSA) for 2 h. The membranes were then incubated with primary antibodies at 4°C overnight. The primary antibodies used were as follows: anti-ZO-1 mouse monoclonal antibody (mAb) (1:500; Cat. No. 33–9100) and anti-occludin mouse mAb (1:5,000; Cat. No. 33–1500) were purchased from Invitrogen (Carlsbad, CA, USA); anti-claudin1 rabbit polyclonal antibody (1:1,000; Cat. No. 13050-1-AP) and anti-vimentin rabbit polyclonal antibody (1:2,000; Cat. No. 10366-1-AP) were purchased from Proteintech (Wuhan, China); anti-phospho-myosin light chain 2 (Thr18/Ser19) rabbit antibody (1:1,000; Cat. No. 3674) and anti-myosin light chain 2 rabbit antibody (1:1,000; Cat. No. 3672) were purchased from Cell Signaling Technology (Beverly, MA, USA); anti-DYKDDDDK (FLAG) tag mouse mAb (1:5,000; Cat. No. AT0022), anti-HA tag mouse mAb (1:5,000; Cat. No. AT0024), anti-GAPDH mouse mAb (1:5,000; Cat. No. AT0002) and anti-beta actin mouse mAb (1:5,000; Cat. No. AT0001) were purchased from Engibody Biotechnology (Milwaukee, WI, USA).

The membranes were washed with TBST and then incubated with HRP-conjugated goat anti-rabbit (1:5,000; Cat. No. AT0097, Engibody Biotechnology) or goat anti-mouse IgG antibody (1:5,000; Cat. No. AT0098, Engibody Biotechnology) at room temperature for 1 h. The membranes were incubated with ECL Femto-Detect Western Blotting Substrate (Engibody Biotechnology) and exposed using a ChemiDoc Touch Imaging System (Bio-Rad), and the resulting images were analyzed using Image Lab software. The images shown are representative of three independent experiments.

### Immunofluorescence

After deparaffinization and antigen retrieval, mouse lung sections were blocked with 3% BSA. For immunostaining of STECs, the cells were fixed in 4% paraformaldehyde and permeabilized in PBS containing 0.1% Triton X-100 for 5 min. Nonspecific protein binding sites were blocked with 3% BSA for 2 h. The coverslips were then incubated with anti-ZO-1 mouse mAb (5 μg/mL, Invitrogen) and anti-occludin mouse mAb (2 μg/mL, Invitrogen) at 4°C overnight. The coverslips were washed three times with PBS and incubated with DyLight 488-conjugated goat anti-mouse IgG antibody (1:400; Cat. No. A23210, Abbkine, Wuhan, China) for 1 h at room temperature. 4’,6-Diamidino-2-phenylindole (DAPI, Beyotime) was used to stain the nuclei. The slides were mounted using ProLong Gold antifade reagent (Invitrogen) and photographed using a laser scanning confocal microscope (LSCM, ZEISS, Germany).

To detect the adhesion of rAtl to STECs, endotoxin free rAtl was added to the STEC monolayer and the plates were incubated at 37°C in a 5% CO_2_ incubator for 3 h. The cells were fixed in cold methanol for 20 min and blocked with 3% BSA for 2 h. They were then incubated with anti-Atl rabbit polyclonal antibody (1:400) (obtained by immunizing rabbits with purified rAtl protein) at 4°C overnight and then with DyLight 488-conjugated goat anti-rabbit IgG antibody (1:400; Cat. No. A23210, Abbkine) at room temperature for 1 h. The nuclei were stained with DAPI. After mounting, the slides were observed and photographed under a fluorescence microscope (ZEISS, Germany).

### Pull-down assay

STECs in a 10 cm Petri dish were lysed with 2 mL of IP buffer (Thermo Fisher Scientific, Waltham, MA, USA). The cell supernatant was obtained by centrifugation (4°C, 13,000 × *g*, 10 min) and used as prey protein. The bait protein, His-tagged Atl, was immobilized on high-affinity Ni-NTA (GenScript, Nanjing, China). The prey protein was incubated with the bait protein immobilized on Ni-NTA at 4°C for 2 h. After three washes with a binding buffer containing 20 mM imidazole, the bait-prey complex was eluted in elution buffer containing 400 mM imidazole. The solution was boiled for 10 min with 5 × SDS-PAGE loading buffer (Beyotime) and subjected to SDS-PAGE. As a negative control, cell lysates were added directly to Ni-NTA without His-tagged Atl or Ni-NTA with His-tag. Following separation in a polyacrylamide gel, the protein bands that were present in the experimental lanes but not in the control lanes were excised and subjected to LC-MS/MS (Shanghai Applied Protein Technology Co., Ltd., China) or Western blot analysis.

A GST pull-down assay was performed to verify the interaction between Atl and vimentin. Briefly, the bait protein, GST or GST-vimentin, was mixed with 50% GST-tag purification resin (Beyotime) at 4°C for 2 h. After washing five washes with PBST, the resins were incubated with His-tagged Atl at 4°C for 2 h with gentle rocking, the bait-prey complex was eluted with elution buffer after six washes. The protein complexes were analyzed by SDS-PAGE followed by immunoblotting with mouse anti-GST tag mAb (1:5,000; Cat. No. K200006M, Solarbio) and rabbit anti-Atl polyclonal antibody (1:1,000).

### Co-IP assay

Atl-pcDNA3-2xFLAG and vimentin-pCAGGS-HA or Bsp-pCAGGS-HA were cotransfected into HEK293T cells. At 48 h after transfection, the cells were lysed with IP buffer containing protease inhibitors and phosphatase inhibitors (Beyotime) and centrifuged at 13,000 × *g* at 4°C for 10 min. The resulting supernatants were incubated with anti-DYKDDDDK (FLAG) tag mouse mAb (1:400; Cat. No. AT0022, Engibody Biotechnology) at 4°C for 2 h. Then, 50% protein-G agarose beads (Beyotime) were added, and the mixture was incubated at 4°C overnight with rotation. The agarose bead conjugates were subsequently washed six times with TBST and boiled in 2 × SDS loading buffer, and the proteins present were detected by Western blot.

### Bacterial two-hybrid assay

A bacterial adenylate cyclase-based two-hybrid system was used to verify the interaction of Atl and vimentin *in vivo*, as described previously [53, 54]. Briefly, Atl-pUT18 and vimentin-pKT25 plasmids or empty vector control were co-transformed into BTH101 reporter cells that lack endogenous adenylate cyclase activity. The transformants were identified on LB plates containing X-Gal (40 μg/mL, Beyotime) and IPTG (0.5 M) or MacConkey plates containing maltose (1%) and IPTG (0.5 M).

### Saturation binding experiment

The saturation binding experiment was performed as described previously with some modification [55]. Microplates (Stripwell, Corning) were coated with vimentin-GST protein (0.1, 1, 2.5, 5, 10, and 20 μg/mL) in carbonate buffer (0.1 M, pH 9.6) (100 μL per well) at 4°C overnight. BSA and GST proteins were coated at the same serial concentrations as the negative control. The next day, the plates were washed with PBST (PBS containing 0.05% Tween-20), blocked with 5% skim milk in PBST at room temperature for 1 h, and incubated with 20 μg/mL rAtl protein in PBS at 4°C overnight. After washing with PBST, the wells were incubated with anti-Atl rabbit polyclonal antibody (1:400) at 37°C for 1 h. HRP-conjugated goat anti-rabbit IgG antibody (1:5,000; Cat. No. AT0097, Engibody Biotechnology) was then applied, and the plates were incubated at 37°C for 1 h. Between each incubation step, the plates were washed three times with PBST. Soluble TMB substrate solution (TIANGEN Biotech (Beijing) Co., China) was added, and the reaction was quenched with 2 M H_2_SO_4_ before reading the absorbance at 450 nm.

### Establishment of a vimentin knockout STEC cell line

Three pairs of sgRNAs targeting the porcine vimentin genome sequence were cloned into the lentiCRISPR v2 vector (Addgene, USA) and cotransfected with pCMV-VSV-G (Addgene) and psPAX2 (Addgene) into HEK293T cells for lentivirus packaging. STECs were infected with lentiviruses at an MOI of 1:1 for 36 h. The on-target efficiency was analyzed by qRT-PCR and Western blot. The on-target efficiency in the control group was set as 0. A single clone was screened in the presence of puromycin (2 μg/mL, Sigma-Aldrich) for 7 d. Western blot and immunofluorescence were used to determine the expression of vimentin.

### Efficacy of inhibitors

STECs in a 10 cm Petri dish were pretreated with 10 μM ML-7 (MCE, China), an MLCK inhibitor, for 1 h. The STECs were then infected with SS2 at an MOI of 50:1. After 3 h, immunofluorescence staining was performed, or membrane proteins were extracted for Western blot detection.

### Data analysis

GraphPad Prism 7 software (La Jolla, CA, USA) was used for data analysis. For all experiments, the data shown represent at least three independent experiments and are presented as the mean ± standard deviation (SD). An unpaired t test was used to analyze the statistical significance of the difference between two data sets that passed normality tests, while a Mann-Whitney test was performed if the data did not pass the normality test. Differences between more than two sets of data were assessed using one-way ANOVA or two-way ANOVA. *P* < 0.05 indicated a significant difference.

## Supporting information

S1 Fig Analysis of the invasion rates of mutant strains. The unpaired t test was used to test the significance of the data. *, *P* < 0.05; **, *P* < 0.01; ***, *P* < 0.001.
(PDF)

S2 Fig Construction of Δ*atl* and CΔ*atl*. (A) Δ*atl* was identified by PCR using primers Δ*atl*-F1/Δ*atl*-R2. (B) Δ*atl* was identified by PCR using primers IN-*atl*-F/IN-*atl*-R. (C) Schematic diagram of the site mutation of CΔ*atl*. (D) PCR detection of the complementary plasmid CΔ*atl*-pSET4S using primers CΔ*atl*-F1/CΔ*atl*-R2. (E) PCR identification of CΔ*atl* using primers CΔ*atl*-F1/CΔ*atl*-R2. M: DL5000 DNA Marker.
(PDF)

S3 Fig Construction of the tracheal epithelial barrier model. (A) Transepithelial electrical resistance (TEER) value of the tracheal epithelial barrier model. (B) FD-4 flux of STECs grown on Transwell inserts. The unpaired t test was used to test the significance of the data. ****, *P* < 0.0001.
(PDF)

S4 Fig Effect of Atl on the TEER of the tracheal epithelial barrier. The ratio of the TEER of STEC monolayers grown on Transwell inserts infected with WT SS2, with the Δ*atl* mutant or with the CΔ*atl* strain at 3 h to the TEER at 0 h was calculated. The data are presented as the mean ± SD of the values obtained in three independent experiments. One-way ANOVA with Dunnett’s multiple comparison test was used to test the significance of the data. ***, *P* < 0.001; ****, *P* < 0.0001.
(PDF)

S5 Fig Growth of WT, Δ*atl*, and CΔ*atl* strains in THY medium (Todd-Hewitt broth w/2% yeast extract). (PDF)

S6 Fig Effect of SS2 infection on TJs. STECs were infected with WT SS2 (MOI 50:1) for the indicated times. (A) qRT-PCR analysis of the effect of SS2 on the transcription of the TJ proteins ZO-1, occludin, and claudin1. (B) Western blot analysis of TJ protein (ZO-1, occludin, and claudin1) levels in whole-cell extracts from STECs. Band intensity relative to the uninfected group was analyzed. The data are presented as the mean ± SD of the values obtained in three independent experiments. Two-way ANOVA with Dunnett’s multiple comparison was used to test the significance of the data. ns, not significant.
(PDF)

S7 Fig Detection of the effect of SS2 on STEC activity by the MTT assay. The data are presented as the mean ± SD of the values obtained in three independent experiments. Statistical analysis was performed using one-way ANOVA with Tukey’s multiple comparison test. ns, not significant.
(PDF)

S8 Fig Effect of Atl on the distribution of claudin1 and occludin in STECs. STECs were infected with WT, Δ*atl*, or CΔ*atl* at an MOI of 50:1. The expression of claudin1 and occludin in the cell membrane of STECs was analyzed by Western blot. The immunoblots shown are representative of the results obtained in three independent experiments.
(PDF)

S9 Fig Effect of the MLCK inhibitor ML-7 on the TEER of the tracheal epithelial barrier. STEC monolayers on Transwell inserts were pretreated with ML-7 or DMSO prior to SS2 infection. The ratio of the TEER of STEC monolayers infected with the WT or Δ*atl* mutant strain at 3 h to the TEER at 0 h was calculated. Data are presented as the mean ± SD of the values obtained in three independent experiments. One-way ANOVA with Tukey’s multiple comparison test was used to test the significance of the data. ****, *P* < 0.0001. ns, not significant.
(PDF)

S10 Fig Establishment of the VIM KO STEC cell line. (A and B) Transcription (qRT-PCR) (A) and protein (Western blot) expression levels (B) of vimentin after 36 h of lentivirus infection in STECs. One-way ANOVA was used to test the significance of the data in A. ****, *P* < 0.0001. (C) Analysis of sgRNA targeting efficiency. (D) Western blot detection of vimentin expression in monoclonal STECs. (E) Detection of vimentin expression in VIM KO STECs by immunofluorescence staining. Scale bar, 20 μm.
(PDF)

S11 Fig Bacterial loads in tissues of Δ*atl*-infected mice. Vim^+/+^ and Vim^-/-^ mice were challenged with 1 × 10^9^ CFUs SS2 Δ*atl* mutant intranasally, and the bacterial loads in the blood (A), lung (B), brain (C), spleen (D) and liver (E) were determined at 24 hpi (n = 5 mice/group).
(PDF)

S12 Fig Conservation of the GBS Bsp-like domain among respiratory pathogens. (A) GBS Bsp-like domain protein homology alignment. (B) Analysis of the evolutionary relationship of GBS Bsp-like domains.
(PDF)

S1 Table Identification of transposon insertion sites in mutant strains with reduced invasion ability. (DOCX)

S2 Table Primers used in this study. (DOCX)

S1 Data Excel spreadsheet containing the numerical data and statistical analysis for Figure panels 1A–1G, 1I–1J, 2A, 2B, 2C, 2D, 2E, 3A, 3B, 3C, 3D, 3E, 4A, 4B, 4C, 5I, 6A, 6B, 6C, 6D, 7A–7E, S1, S3A, S3B, S4, S6A, S6B, S7, S9, and S11A–S11E. (XLSX)
