# Supplementary figures and images for: Streptococcal autolysin promotes dysfunction of swine tracheal epithelium by interacting with vimentin
Source: PLoS Pathog. 2022 Aug 3;18(8):e1010765. doi: 10.1371/journal.ppat.1010765 (PMC9377611; doi:10.1371/journal.ppat.1010765)

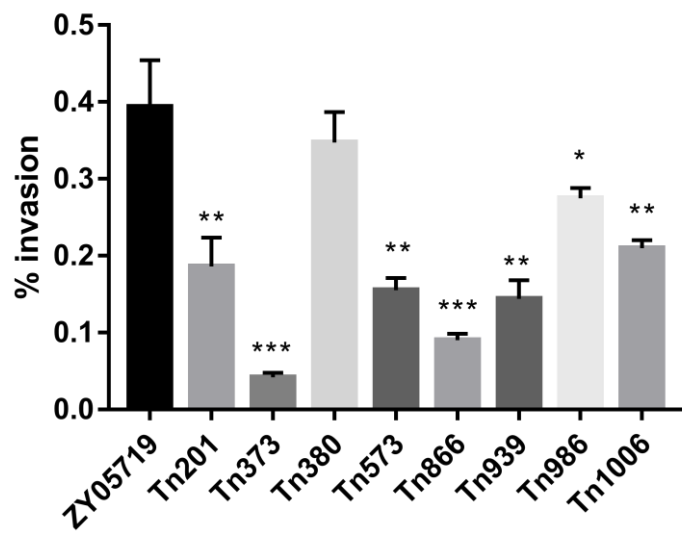

Supplement: S1 Fig — The unpaired t test was used to test the significance of the data. *, P < 0.05; **, P < 0.01; ***, P < 0.001. (PDF) [file ppat.1010765.s001.pdf]

**A**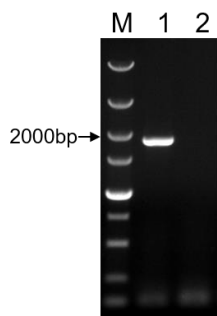**B**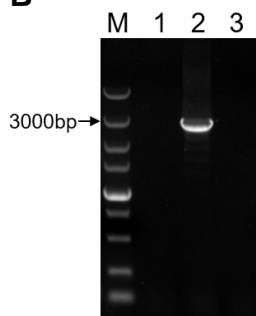**C**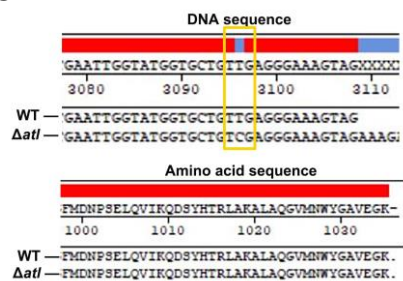**D**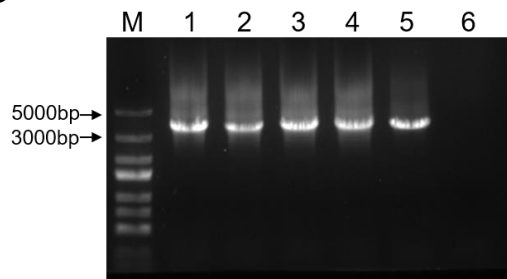**E**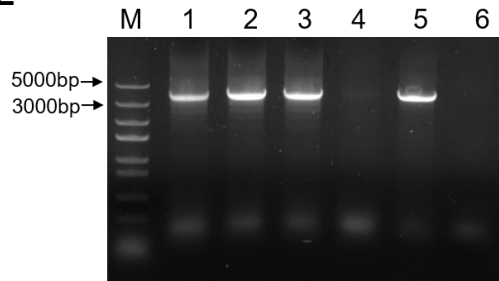

Supplement: S2 Fig — (A) Δatl was identified by PCR using primers Δatl-F1/Δatl-R2. (B) Δatl was identified by PCR using primers IN-atl-F/IN-atl-R. (C) Schematic diagram of the site mutation of CΔatl. (D) PCR detection of the complementary plasmid CΔatl-pSET4S using primers CΔatl-F1/CΔatl-R2. (E) PCR identification of CΔatl using primers CΔatl-F1/CΔatl-R2. M: DL5000 DNA Marker. (PDF) [file ppat.1010765.s002.pdf]

**A**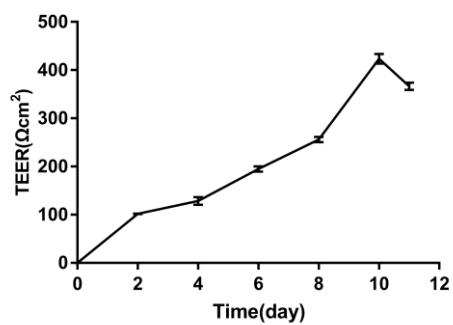**B**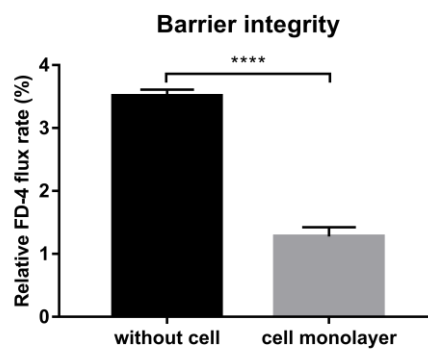

Supplement: S3 Fig — (A) Transepithelial electrical resistance (TEER) value of the tracheal epithelial barrier model. (B) FD-4 flux of STECs grown on Transwell inserts. The unpaired t test was used to test the significance of the data. ****, P < 0.0001. (PDF) [file ppat.1010765.s003.pdf]

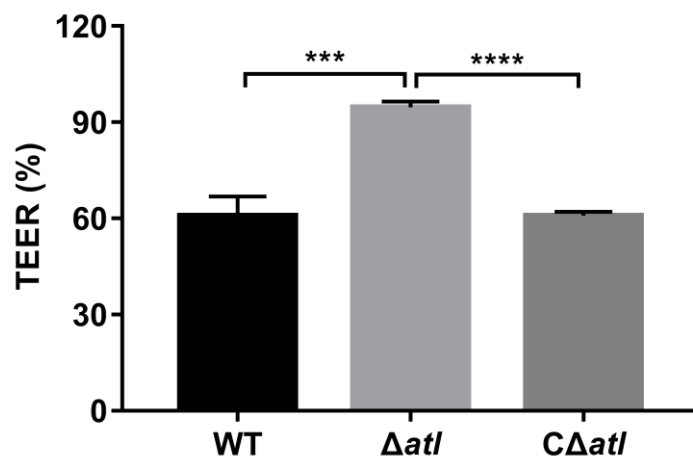

Supplement: S4 Fig — The ratio of the TEER of STEC monolayers grown on Transwell inserts infected with WT SS2, with the Δatl mutant or with the CΔatl strain at 3 h to the TEER at 0 h was calculated. The data are presented as the mean ± SD of the values obtained in three independent experiments. One-way ANOVA with Dunnett’s multiple comparison test was used to test the significance of the data. ***, P < 0.001; ****, P < 0.0001. (PDF) [file ppat.1010765.s004.pdf]

Growth curve

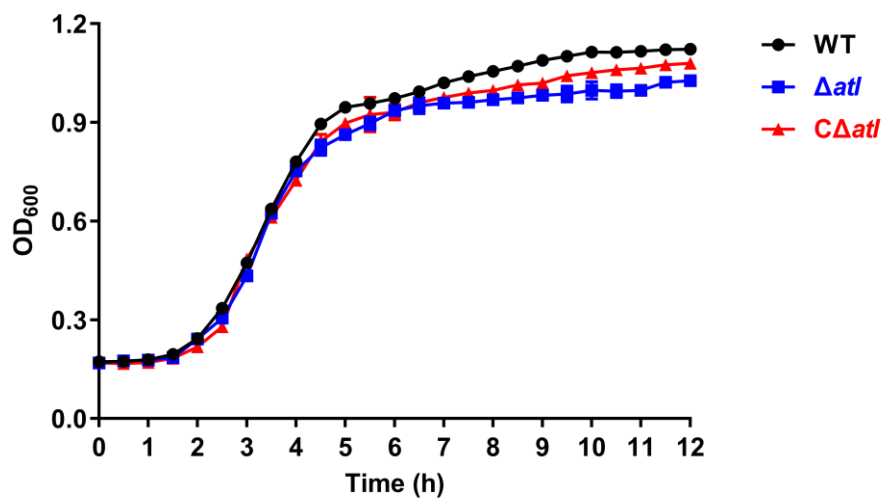

Supplement: S5 Fig — (PDF) [file ppat.1010765.s005.pdf]

**A**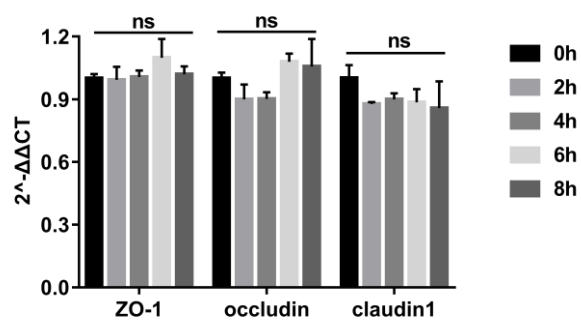**B**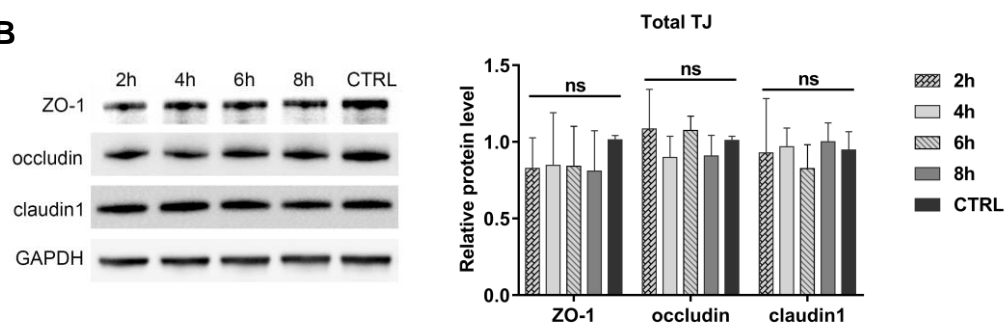

Supplement: S6 Fig — STECs were infected with WT SS2 (MOI 50:1) for the indicated times. (A) qRT-PCR analysis of the effect of SS2 on the transcription of the TJ proteins ZO-1, occludin, and claudin1. (B) Western blot analysis of TJ protein (ZO-1, occludin, and claudin1) levels in whole-cell extracts from STECs. Band intensity relative to the uninfected group was analyzed. The data are presented as the mean ± SD of the values obtained in three independent experiments. Two-way ANOVA with Dunnett’s multiple comparison was used to test the significance of the data. ns, not significant. (PDF) [file ppat.1010765.s006.pdf]

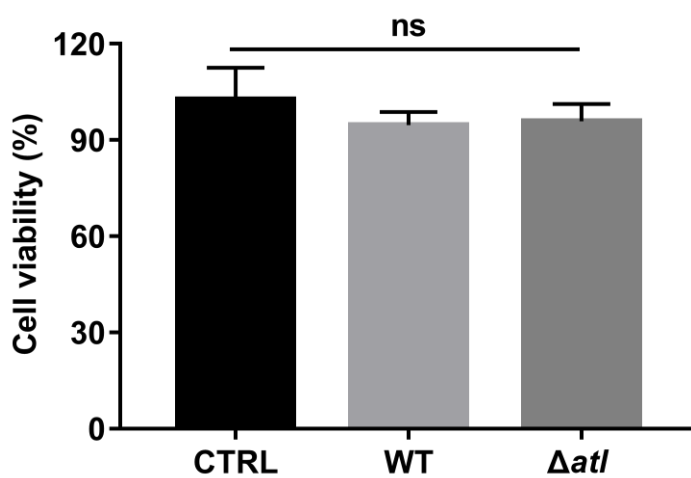

Supplement: S7 Fig — The data are presented as the mean ± SD of the values obtained in three independent experiments. Statistical analysis was performed using one-way ANOVA with Tukey’s multiple comparison test. ns, not significant. (PDF) [file ppat.1010765.s007.pdf]

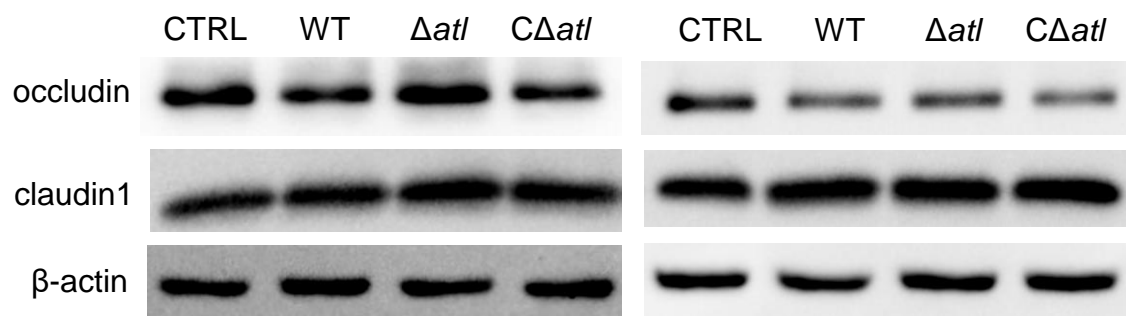

Supplement: S8 Fig — STECs were infected with WT, Δatl, or CΔatl at an MOI of 50:1. The expression of claudin1 and occludin in the cell membrane of STECs was analyzed by Western blot. The immunoblots shown are representative of the results obtained in three independent experiments. (PDF) [file ppat.1010765.s008.pdf]

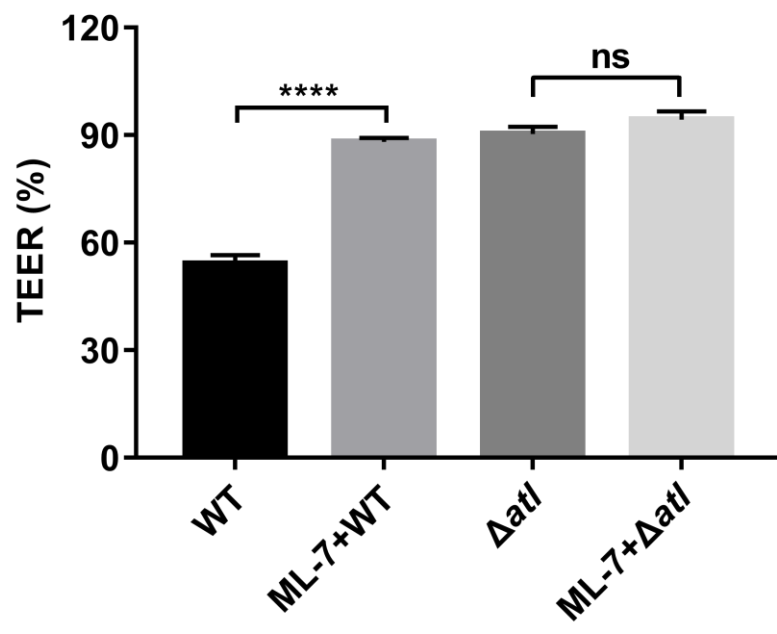

Supplement: S9 Fig — STEC monolayers on Transwell inserts were pretreated with ML-7 or DMSO prior to SS2 infection. The ratio of the TEER of STEC monolayers infected with the WT or Δatl mutant strain at 3 h to the TEER at 0 h was calculated. Data are presented as the mean ± SD of the values obtained in three independent experiments. One-way ANOVA with Tukey’s multiple comparison test was used to test the significance of the data. ****, P < 0.0001. ns, not significant. (PDF) [file ppat.1010765.s009.pdf]

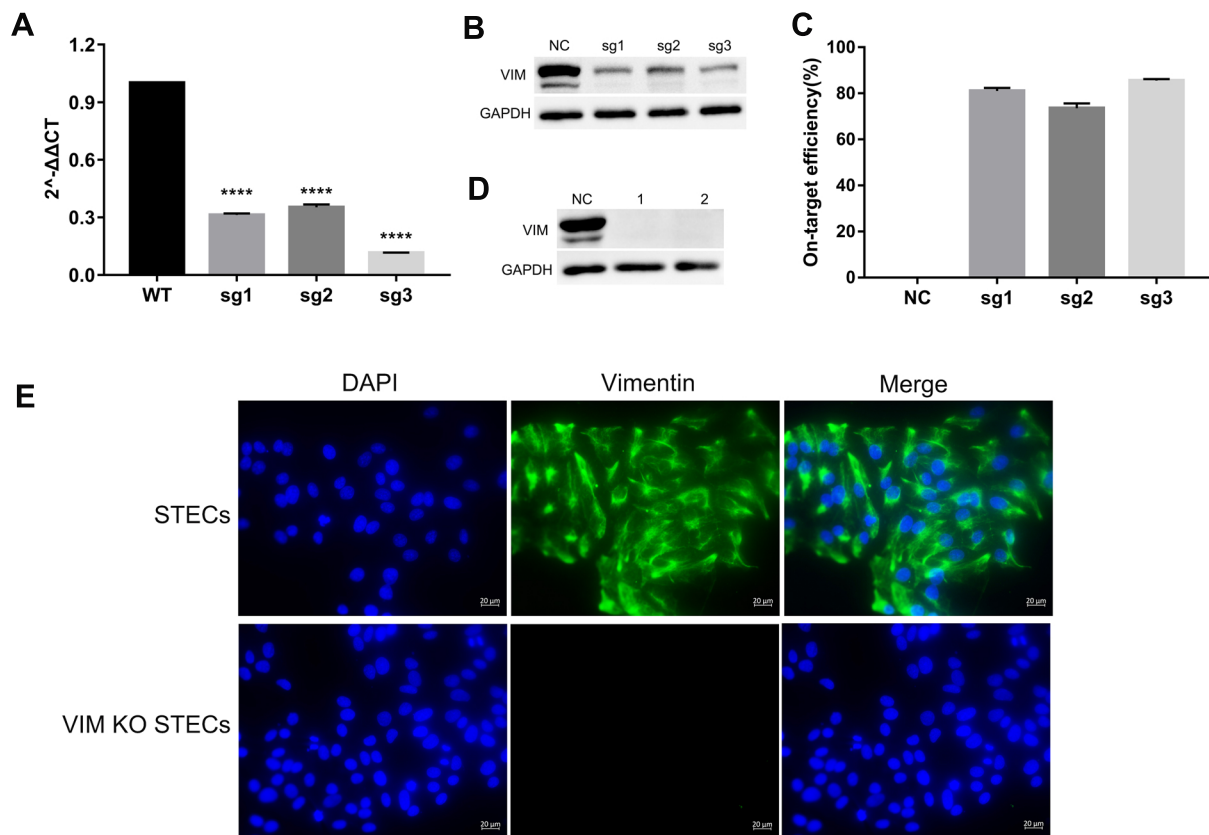

Supplement: S10 Fig — (A and B) Transcription (qRT-PCR) (A) and protein (Western blot) expression levels (B) of vimentin after 36 h of lentivirus infection in STECs. One-way ANOVA was used to test the significance of the data in A. ****, P < 0.0001. (C) Analysis of sgRNA targeting efficiency. (D) Western blot detection of vimentin expression in monoclonal STECs. (E) Detection of vimentin expression in VIM KO STECs by immunofluorescence staining. Scale bar, 20 μm. (PDF) [file ppat.1010765.s010.pdf]

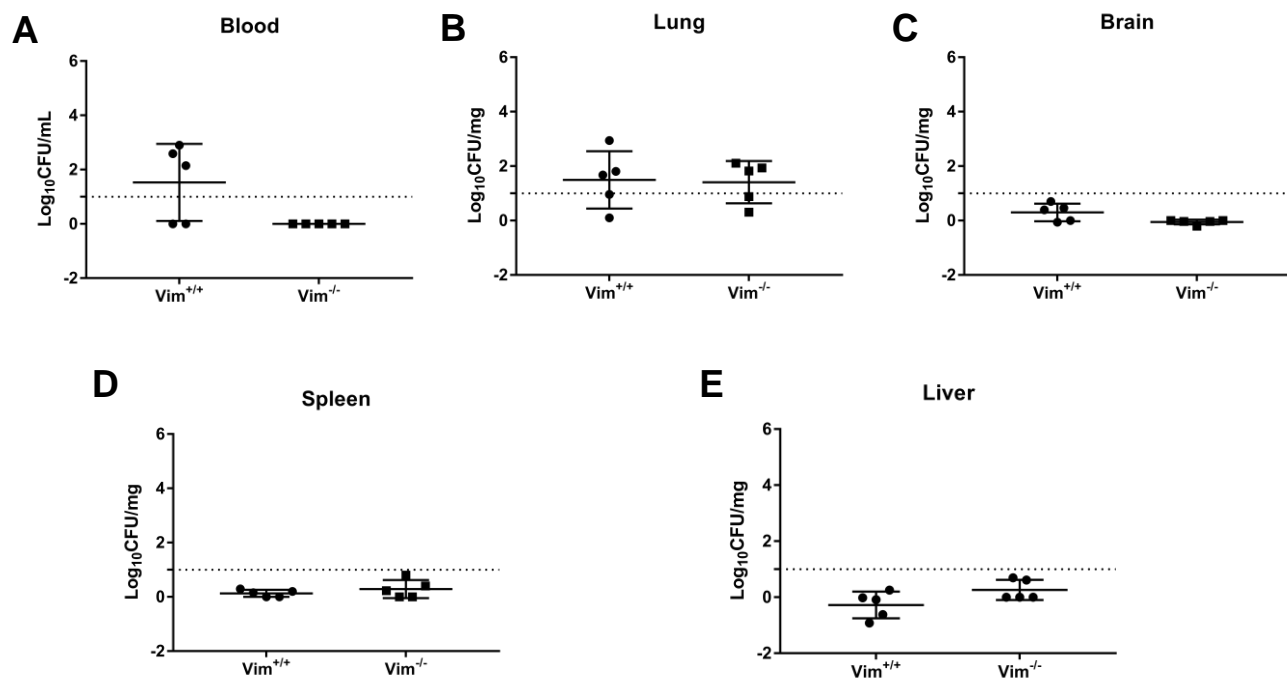

Supplement: S11 Fig — Vim+/+ and Vim-/- mice were challenged with 1 × 10^9^ CFUs SS2 Δatl mutant intranasally, and the bacterial loads in the blood (A), lung (B), brain (C), spleen (D) and liver (E) were determined at 24 hpi (n = 5 mice/group). (PDF) [file ppat.1010765.s011.pdf]
