# Supplementary material for: Streptococcal autolysin promotes dysfunction of swine tracheal epithelium by interacting with vimentin
Source: PLoS Pathog. 2022 Aug 3;18(8):e1010765. doi: 10.1371/journal.ppat.1010765 (PMC9377611; doi:10.1371/journal.ppat.1010765)
Supplement: S12 Fig — (A) GBS Bsp-like domain protein homology alignment. (B) Analysis of the evolutionary relationship of GBS Bsp-like domains. (PDF) [file ppat.1010765.s012.pdf]

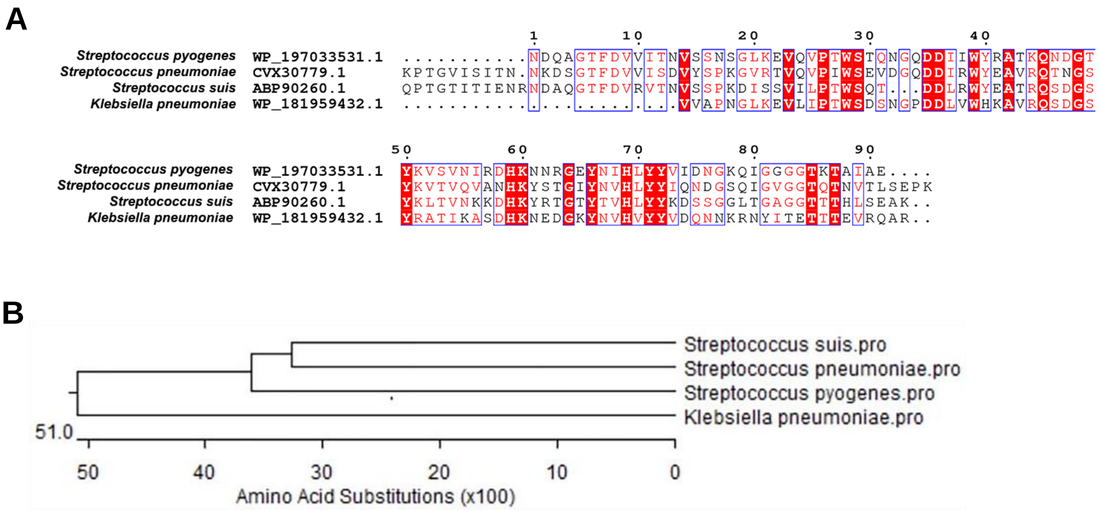
A
1 10 20 30 40
Streptococcus pyogenes WP_197033531.1 ...........NDQAGTFDVVITNVSSNSGLKEVQVPTWSTQNGQDDIIWYRATKQNDGT
Streptococcus pneumoniae CVX30779.1 KPTGVISITN.NKDSGTFDVVISDVYSPKGVRTVQVPIWSEVDGQDDIRWYEAVRQTNGS
Streptococcus suis ABP90260.1 QPTGTITIENRNDAQGTFDVRVTNVSSPKDISSVILPTWSQT...DDLRWYEATRQSDGS
Klebsiella pneumoniae WP_181959432.1 ........................VVAPNGLKEVLIPTWSDSNGPDDLVWHKAVRQSDGS
50 60 70 80 90
Streptococcus pyogenes WP_197033531.1 YKVSVNIRDHKNNRGEYNIHLYYVIDNGKQIGGGTKTAIAE....
Streptococcus pneumoniae CVX30779.1 YKVTVQVANHKYSTGIYNVHLYYIQNDGSQIGVGGTQTNVTLSEPK
Streptococcus suis ABP90260.1 YKLTVNKKDHKYRTGTYTVHLYYKDSSGGLTGAGGTTTHLSEAK..
Klebsiella pneumoniae WP_181959432.1 YRATIKASDHKNEDGKYNVHVYYVDQNNKRNYITETTTEVRQAR..
B
Streptococcus suis.pro
Streptococcus pneumoniae.pro
Streptococcus pyogenes.pro
Klebsiella pneumoniae.pro
51.0
50 40 30 20 10 0
Amino Acid Substitutions (x100)
