# Supplementary material for: Streptococcal autolysin promotes dysfunction of swine tracheal epithelium by interacting with vimentin
Source: PLoS Pathog. 2022 Aug 3;18(8):e1010765. doi: 10.1371/journal.ppat.1010765 (PMC9377611; doi:10.1371/journal.ppat.1010765)
Supplement: S1 Table — (DOCX) [file ppat.1010765.s013.docx]

**S1 Table.** Identification of transposon insertion sites in mutant strains with reduced invasion abilities.

| **Mutant** | **gene locus** | **protein** |
| --- | --- | --- |
| **Tn201** | ZY05719_RS10335 | PDZ domain-containing protein（HtrA） |
| **Tn205** | ZY05719_RS08865 | Glutamine ABC transporter substrate-binding protein/permease |
| **Tn280** | ZY05719_RS08990 | sn-glycerol-3-phosphate ABC transporter ATP-binding protein UgpC |
| **Tn373** | ZY05719_RS06135 | N-acetylmuramoyl-L-alanine amidase (Atl) |
| **Tn380** | ZY05719_RS03220 | YitT family protein |
| **Tn573** | ZY05719_RS02785 | tyrosine protein phosphatase(Cps2D) |
| **Tn689** | ZY05719_RS08870 | glutamine ABC transporter substrate-binding protein |
| **Tn866** | ZY05719_RS07585 | Methyl-accepting chemotaxis-like domain-containing protein |
| **Tn939** | ZY05719_RS06135 | cell wall hydrolase/autolysin |
| **Tn957** | ZY05719_RS07040 | PTS beta-glucoside transporter subunit IIABC |
| **Tn966** | ZY05719_RS02075 | Stp1/IreP family PP2C-type Ser/Thr phosphatase |
| **Tn986** | ZY05719_RS08190 | AI-2E family transporter |
| **Tn1006** | ZY05719_RS05415 | MarR family transcriptional regulator |
