# Supplementary material for: Streptococcal autolysin promotes dysfunction of swine tracheal epithelium by interacting with vimentin
Source: PLoS Pathog. 2022 Aug 3;18(8):e1010765. doi: 10.1371/journal.ppat.1010765 (PMC9377611; doi:10.1371/journal.ppat.1010765)
Supplement: S2 Table — (DOCX) [file ppat.1010765.s014.docx]

**S2 Table.** Primers used in this study.

| **Primer name** | **Sequence(5'-3' orientation)** | **Function** |
| --- | --- | --- |
| **Δ*atl* -F1** | TGTAAAACGACGGCCAGTGAATTCCCCTTTAGTGATTTGGTC | Upstream flanking sequence of *atl* |
| **Δ*atl* -R1** | TGAGAAATATAGTTGTTTTTATTGCAGGTACAGGTATTG | Upstream flanking sequence of *atl* |
| **Δ*atl* -F2** | CAATACCTGTACCTGCAATAAAAACAACTATATTTCTCA | Downstream flanking sequence of *atl* |
| **Δ*atl* -R2** | AAGCTTGCATGCCTGCAGGTCGACATATTCAAGCTAAAATGACTA | Downstream flanking sequence of *atl* |
| **IN-*atl*-F** | ACACCTTGTGCCAATGCT | Inside region of *atl* |
| **IN-*atl*-R** | GTCGTTGATGAGGCTGGT | Inside region of *atl* |
| **CΔ*atl*-F1** | CAGTCACGACGTTGTAAAACGACGGCCAGTGAATTCCCCTTTAGTGATTTGGTC |  |
| **CΔ*atl*-R1** | TTGAAGATCTGGTTAAACAG TGTCGAGGGAAAGTAGAAA |  |
| **CΔ*atl*-F2** | TTTCTACTTTCCCTCGACA CTGTTTAACCAGATCTTCAA |  |
| **CΔ*atl*-R2** | CATGATTACGCCAAGCTTGCATGCCTGCAGGTCGACATATTCAAGCTAAAATGACTA |  |
| **EAtl-F** | GAGCTCGGTACCCTCGAGGGATCCGATATGGTAAATGCCCCATCTAGTC | Prokaryotic expression of Atl protein |
| **EAtl-R** | TCTAGACTGCAGGTCGACAAGCTTCTACTTTCCCTCAACAGCACCATAC | Prokaryotic expression of Atl protein |
| **Atl-pcDNA3-2×FLAG-F** | GGTCACCCAGGATCCCCGGAATTCGATATGGTAAATGCCCCATC | Eukaryotic expression of Atl |
| **Atl-pcDNA3-2×FLAG-R** | GGGCCCTCTAGATGCATGCTCGAGCTACTTTCCCTCAACAGCAC | Eukaryotic expression of Atl |
| **Atl- pAcGFP1-C-F** | CCCAAGCTTGATATGGTAAATGCCCCATC | Eukaryotic expression of Atl |
| **Atl- pAcGFP1-C-R** | CGCGGATCCCTACTTTCCCTCAACAGCAC |  |
| **VIM-pCAGGS-HA-F** | GATGTTCCAGATTACGCTGAATTC ATGTCCACCAGGACCGTGT | Eukaryotic expression of vimentin |
| **VIM-pCAGGS-HA-R** | TTAATTAAGATCTGCTAGCTCGAG TTATTCCAGATCATCGTGAT |  |
| **VIM-pAcGFP1-C-F** | CCCAAGCTTTCCACCAGGACCGTGTCCTC | Eukaryotic expression of vimentin |
| **VIM-pAcGFP1-C-R** | CGCGGATCCTTATTCCAGATCATCGTGAT |  |
| **VIM-pGEX-6p-1 F** | CGCGGATCCATGTCCACCAGGACCGTGT | Prokaryotic expression of vimentin protein |
| **VIM-pGEX-6p-1 R** | CCGGAATTCTTATTCCAGATCATCGTGATGC |  |
| **Atl-pUT18 F** | CCCAAGCTTGGTAAATGCCCCATCTAGTCA |  |
| **Atl-pUT18 R** | CGCGGATCC TC CTTTCCCTCAACAGCACCAT |  |
| **VIM-pKT25 F** | CGCGGATCCCATGTCCACCAGGACCGTGT |  |
| **VIM-pKT25 R** | CCGGAATTCTTAGTTATTATTCCAGATCATCGTGATGC |  |
| **VIM sgRNA1-F** | CACCGTCGGCCGGCTAGCGGTGCCA | Targeting sequence for vimentin knockout |
| **VIM sgRNA1-R** | AAACTGGCACCGCTAGCCGGCCGAC | Targeting sequence for vimentin knockout |
| **VIM sgRNA2-F** | CACCGGCCGCCGAACATTCTGCGGT | Targeting sequence for vimentin knockout |
| **VIM sgRNA2-R** | AAACACCGCAGAATGTTCGGCGGCC | Targeting sequence for vimentin knockout |
| **VIM sgRNA3-F** | CACCGTCCTACCGCAGAATGTTCGG | Targeting sequence for vimentin knockout |
| **VIM sgRNA3-R** | AAACCCGAACATTCTGCGGTAGGAC | Targeting sequence for vimentin knockout |
| **qGAPDH F** | TGGTCACCAGGGCTGCTT |  |
| **qGAPDH R** | CATGTAGTGGAGGTCAATGAAGG |  |
| **qVIM-F** | CAGATCCAGGAACAGCACGT | qPCR of vimentin |
| **qVIM-R** | GAGAGGTCGGCAAACTTGGA | qPCR of vimentin |
| **qMLCK-F** | AAACCGCTTGGACTGCAC | qPCR of MLCK |
| **qMLCK-R** | ACTTTTTCATCCGGTCCTTG | qPCR of MLCK |
